# ERBB1/EGFR and JAK3 Tyrosine Kinases as Potential Therapeutic Targets in High-Risk Multiple Myeloma

**DOI:** 10.3390/onco2040016

**Published:** 2022-10-14

**Authors:** Fatih M. Uckun, Sanjive Qazi

**Affiliations:** 1 Immuno-Oncology Program, Ares Pharmaceuticals, St. Paul, MN 55110, USA; 2 Division of Hematology-Oncology, Department of Pediatrics and Developmental Therapeutics Program, Norris Comprehensive Cancer Center, University of Southern California Keck School of Medicine (USC KSOM), Los Angeles, CA 90027, USA

**Keywords:** ERBB1, EGFR, JAK3, tyrosine kinase, multiple myeloma

## Abstract

Our main objective was to identify abundantly expressed tyrosine kinases in multiple myeloma (MM) as potential therapeutic targets. We first compared the transcriptomes of malignant plasma cells from newly diagnosed MM patients who were risk-categorized based on the patient-specific EMC-92/SKY-92 gene expression signature values vs. normal plasma cells from healthy volunteers using archived datasets from the HOVON65/GMMG-HD4 randomized Phase 3 study evaluating the clinical efficacy of bortezomib induction/maintenance versus classic cytotoxic drugs and thalidomide maintenance. In particular, ERBB1/EGFR was significantly overexpressed in MM cells in comparison to normal control plasma cells, and it was differentially overexpressed in MM cells from high-risk patients. Amplified expression of EGFR/ERBB1 mRNA in MM cells was positively correlated with increased expression levels of mRNAs for several DNA binding proteins and transcription factors with known upregulating activity on EGFR/ERBB1 gene expression. MM patients with the highest ERBB1/EGFR expression level had significantly shorter PFS and OS times than patients with the lowest ERBB1/EGFR expression level. High expression levels of EGFR/ERBB1 were associated with significantly increased hazard ratios for unfavorable PFS and OS outcomes in both univariate and multivariate Cox proportional hazards models. The impact of high EGFR/ERBB1 expression on the PFS and OS outcomes remained significant even after accounting for the prognostic effects of other covariates. These results regarding the prognostic effect of EGFR/ERBB1 expression were validated using the MMRF-CoMMpass RNAseq dataset generated in patients treated with more recently applied drug combinations included in contemporary induction regimens. Our findings provide new insights regarding the molecular mechanism and potential clinical significance of upregulated EGFR/ERBB1 expression in MM.

## Simple Summary

Several tyrosine kinase inhibitors have gone through a meticulous drug development process and have been approved for specific oncologic indications. These precision medicines could be re-purposed as new drugs for diseases beyond their approved target indications if the expression of their specific targets is not limited to the cancer types they are approved for. In the current study, we examined the expression of a broad panel of tyrosine kinase genes in malignant cells from multiple myeloma patients. We identified some tyrosine kinases that are expressed at significantly higher levels in malignant plasma cells from multiple myeloma patients than normal plasma cells. One of these tyrosine kinases is EGFR/ERBB1. Interestingly, multiple myeloma patients with the highest EGFR/ERBB1 expression had worse survival outcomes. Therefore, EGFR/ERBB1 inhibitors may be particularly meaningful as adjuncts of available standard treatment options for these patients. The new insights provided by our study warrant further investigation of the clinical potential of tyrosine kinase inhibitors of EGFR/ERBB1 in treatment of multiple myeloma.

## 1. Introduction

Multiple myeloma (MM) is the second most common B-lineage lymphoid malignancy [1,2]. Recent therapeutic advances, including the incorporation of immunomodulatory drugs (IMIDs) and proteasome inhibitors (PI) into frontline and salvage regimens, have significantly improved the survival outcome of multiple myeloma (MM) [2], but treatment failures and relapse are still common due to inherent and/or acquired cancer drug resistance coupled with an immunosuppressive tumor microenvironment facilitating the immune evasion by drug-resistant MM clones [2-12]. MM patients with triple-class-refractory disease whose malignant clones are characterized by resistance to PIs, IMiDs, and monoclonal antibodies have an overall survival of <6 months emphasizing the urgency of identifying effective strategies to overcome triple-class cancer drug resistance [3,4]. Due to the disappointingly poor outcome of MM patients following a relapse on contemporary frontline regimens, effective treatment strategies for relapsed/refractory MM are urgently needed [1,2,4,5,8]. Personalized therapy platforms, such as precision medicines, bispecific antibodies, CAR-T cells, and antibody therapeutics continue to contribute to improved survival outcomes and thereby change the therapeutic landscape for MM [2,6-9]. Promising clinical data with anti-CD38 monoclonal antibodies daratumumab and isatuximab and the anti-signaling lymphocyte activation marker F7 (SLAMF7) antibody elotuzumab, CD3-engaging bispecific antibodies redirecting T-cells to MM targets such as CD38, the orphan G protein-coupled receptor GPRC5D, or the B-cell maturation antigen (BCMA)/CD269, as well as chimeric antigen receptor (CAR)-T cells or natural killer (NK) cells have contributed to a renewed hope regarding the potential of overcoming cancer drug resistance in MM [2]. Nevertheless, these innovative platforms have their own shortcomings causing resistance of MM clones and/or potentially life-threatening side effects, such as cytokine release syndrome (CRS) [2].

Several putative driver mutations in MM cells are considered druggable and some have been targeted with serine/threonine kinase inhibitors developed as precision medicines [2]. Examples include the development of the CDK4/6 inhibitor Palbociclib for MM with CCND1 and CDKN2C mutations [13], the development of the RAF kinase inhibitor Encorafenib (LGX818; RAF kinase inhibitor) and the MEK inhibitor Binimetinib (MEK162) for MM with BRAF V600E/K mutation [2,13,14]. However, there is insufficient information regarding the clinical potential of tyrosine kinase inhibitors (TKI) as possible components of future personalized treatment strategies for cancer drug-resistant MM.

Protein tyrosine kinases (PTK) play critical roles in normal lymphohematopoiesis, and they have also been implicated as oncoproteins in the development of B-lineage lymphoid malignancies, including leukemias, lymphomas, and MM [15-17]. The main goal of the present study was to identify abundantly expressed tyrosine kinases of MM cells as potential therapeutic targets with an emphasis on the relative gene expression levels of for 21 PTK, including ERBB1/epidermal growth factor receptor (EGFR), ERBB2, ERBB3, JAK1, JAK2, JAK3, TYK2, FGR, FLT3, FYN, HCK, LCK, LYN, MERTK, SRC, BLK, BMX, BTK, PTK2, SYK, TEC. The main finding of our study was differentially upregulated expression of EGFR/ERBB1 in MM cells vs. normal plasma cells which was associated with upregulated and correlated expression of several DNA binding proteins, which provides a cogent explanation for the observed EGFR/ERBB1 upregulation. The previously unknown differentially augmented expression of EGFR/ERBB1 in MM cells, especially in high-risk patients, suggests that it could serve as a therapeutic target for already approved EGFR/ERBB1 inhibitors. Intriguingly, transcript- level high EGFR/ERBB1 expression was associated with unfavorable PFS and OS outcomes in both univariate and multivariate Cox proportional hazards models. Therefore, high ERBB1/EGFR expression may also have clinical potential as a prognostic biomarker.

## 2. Materials and Methods

### 2.1. Data Normalization for Multiple Myeloma (MM) Samples

We downloaded raw CEL files from 3 datasets deposited in the NCBI repository (GSE171739, GSE19784, and GSE26760 from https://www.ncbi.nlm.nih.gov/geo/ (accessed on 24 September 2022)) to create a working database. We performed a probeset level normalization procedure to enable comparisons of the expression levels of specific genes in CD138^+^ purified malignant plasma cells from high-risk MM patients versus normal CD138^+^ purified normal plasma cells, as well as CD138^+^, purified malignant plasma cells from standard-risk MM patients. The risk categories were based on the patients’ EMC-92/ SKY-92 gene signature profiles [18-21]; we used the published SKY-92 scores to identify high-risk and standard-risk MM patients [18] in our working database. Our working database included normalized data on purified normal plasma cell samples from healthy volunteers (N = 7; GSE171739), purified malignant plasma cell samples from newly diagnosed high-risk MM patients (N = 63; GSE19784) and purified malignant plasma cell samples from newly diagnosed standard-risk MM patients (N = 219; GSE19784). GSE26760 is comprised of samples obtained from the Multiple Myeloma Research Consortium (MMRC) reference collection that were used solely for initial data normalization and quality control of the working data base.

The raw CEL files were pre-processed for batch normalization by utilizing Aroma Affymetrix statistical packages (authored and maintained by Henrik Bengtsson, Dept of Epidemiology & Biostatistics, UCSF (prev. Dept of Statistics, UC Berkeley); aroma.affymetrix_3.2.0, aroma.core_3.2.2, aroma.light_3.24.0, affxparser_1.66.0) ran in R-studio environment (RStudio 2021.09. running with R version 4.1.2 (R Foundation for Statistical Computing, Vienna, Austria. (1 November 2021)) as previously described [22]. The PM signals were quantified using Robust Multiarray Analysis (RMA) in a 3-step process including RMA background correction, quantile normalization, and summarization by a log additive model of probes in a probeset across these samples (RmaPlm method adapted in Aroma Affymetrix). All expression values were log_2_-scaled for visualization in cluster figures. Sample annotations and patient group assignments were obtained from data files in the GEO repository (https://www.ncbi.nlm.nih.gov/geo/, accessed on 24 September 2021): GSE19784_series_matrix.txt.gz, GSE26760_series_matrix.txt.gz and GSE171739_series_matrix. txt.gz. and then accessed using the programming utility, GEOquery_2.62.1 and stringr_1.4.0, implemented in the R environment for samples with SKY-92 score determinations. Gene symbol annotation for each probeset was obtained from the database provided by the Bioconductor repository for R software (http://www.bioconductor.org/ (accessed on 8 July 2022)) (hgu133plus2.db).

Expression values calculated from signals on the Affymetrix platform (log_2_ RMA) were compared to the ranked mean level of the 100 least expressed transcripts across the 7 control samples. The mean and standard deviation values were used to determine the upper 95% confidence level to define the detection limit for the presence of specific transcripts in the MM samples. Box-plots superimposed on the dot plots were used to visualize the gene expression levels in the MM samples compared to the defined detection limit for the two EGFR/ERBB1 probesets: EGFR/ERBB1_1565483_at and EGFR/ERBB1_1565484_x_at.

### 2.2. Statistical Methods for Differential Gene Expression

Our analyses for CD138^+^ purified MM cells and CD138^+^ normal plasma cells from healthy subjects focused on expression levels of the plasma cell MM marker and biotherapy target TNFRSF17/BCMA and 21 PTK, including ERBB1/EGFR, ERBB2, ERBB3, JAK1, JAK2, JAK3, TYK2, FGR, FLT3, FYN, HCK, LCK, LYN, MERTK, SRC, BLK, BMX, BTK, PTK2, SYK, TEC (represented by a total of 65 probesets). Plasma cells were purified by positive magnetic cell sorting selection with CD138 magnetic microbeads (Miltenyi Biotec B.V.) [18,19].

Probesets representing EGFR/ERBB1 were correlated to probesets representing 20 transcription factor genes; IRF1, JUN/ AP-1, SP1, GCFC2/TCF9, TP53, TEAD2/ETF, TBP, POU6F2/RPF-1, FOS/AP-1, FOSB/AP-1, FOSL1/ Fra-1/AP-1, FOSL2/ FRA-2/AP-1, JUND/AP-1, CTNNB1, LEF1, TCF7, TCF7L1, TCF7L2, HOXB5, JUNB/AP-1 across 282 MM patients evaluated on the Affymetrix gene chip (60 probesets in total). Pairwise correlation coefficients were determined for all 60 × 60 (3600 comparisons) probeset combinations and visualized on a heatmap color coded for positive correlations (red = +1) to negative correlations (blue = −1). The clustering algorithm identified co-regulated sets of probesets using the statistical package ggcorrplot_0.1.3 implemented in R. *T*-test was used to test the null hypothesis that the correlation coefficient was equal to zero. Significant correlations were identified for *p*-values less than 0.05 and false discovery rate (FDR) less than 0.10. 2302 significant *p*-values were obtained in this analysis (FDR = 0.078).

Mixed Model ANOVAs implemented in the R version 4.1.2 (1 November 2021) ran in RStudio 2021.09.0 Build 351 environment, as previously described [22,23]. We investigated differential gene expression levels in purified CD138^+^ malignant plasma cells high-risk and standard-risk MM patients as well as purified normal plasma cells from healthy subjects. The statistical model (built using lmerTest_3.1-3 and lme4_1.1-27.1 statistical packages implemented in RStudio) controlled for variation from 2 fixed factors for “probeset” and “diagnostic group”. The variation from the interaction term, “probeset × diagnostic group”, was used to calculate the least square means and standard error values to pairwise comparisons of malignant vs. normal plasma cells and malignant plasma cells from high-risk vs. standard-risk MM patients. Gene chip-togene chip variation was accounted for by the random factor optimized utilizing the REML criterion (fits a variance-component model by residual (or restricted) maximum likelihood). The error term calculated for the interaction term was utilized to calculate statistical significance for linear contrasts performed between comparison groups for each probeset using emmeans_1.7.0 and lsmeans_2.30-0 statistical packages. For each set of comparisons for 65 probesets, we calculated the FDR and the 0.05 significance level and then adjusted the significance level to control for FDR [24]. *p*-values less than 0.05 and FDR less than 0.1 were deemed significant.

To determine the signal detection threshold for Affymetrix probesets, expression values calculated from signals on the Affymetrix platform (log_2_ RMA) were compared to the ranked mean level of the 100 least expressed genes across the 7 control samples. The mean and standard deviation values were used to determine the upper 95% confidence level to define the detection limit for the expression of genes in the MM samples. Box-plots superimposed on the dot plots were used to visualize the gene expression levels in the MM samples compared to the defined detection limit for gene expression using the two EGFR/ERBB1 probesets: EGFR/ERBB1_1565483_at and EGFR/ERBB1_1565484_x_at.

### 2.3. Hierarchical Clustering Analysis

We used a two-way hierarchical clustering technique to organize expression patterns such that sample and probesets displaying similar expression profiles were grouped together using the average distance metric (default Euclidean distance implemented using the heatmap.2 function in the R package gplots_3.1.1), as previously described in detail [25,26].

### 2.4. Overall Survival and Progression-Free Survival Analysis

Archived survival data from newly diagnosed, transplant-eligible patients with newly diagnosed MM from the Dutch-Belgian Cooperative Trial Group for Hematology Oncology Group-65/German-speaking Myeloma Multicenter Group-HD4 (HOVON-65/GMMG-HD4) randomized clinical trial (ISRCTN64455289) were combined with gene expression data (GSE19784) to evaluate the impact of ERBB1/EGFR gene upregulation on progression-free survival (PFS) and overall survival (OS) of the MM patients. Patients enrolled in the HOVON-65/GMMG-HD4 study received vincristine-based (viz.: 3 cycles of vincristine 0.4 mg intravenously on days 1–4, doxorubicin 9 mg/m^2^ intravenously on days 1–4, and dexamethasone 40 mg orally on days 1–4, 9–12, and 17–20 or bortezomib-based (viz.: bortezomib 1.3 mg/m^2^ was given intravenously on days 1, 4, 8, and 11, doxorubicin 9 mg/m^2^ intravenously on days 1–4, and dexamethasone 40 mg orally on days 1–4, 9–12, and 17–20) standard induction chemotherapy After induction therapy, patients received one (HOVON-65) or two (GMMG-HD4) cycles of high-dose melphalan (200 mg/m^2^ intravenously) with autologous stem-cell rescue followed by maintenance treatment with thalidomide (50 mg per day orally; group assigned to vincristine-based induction treatment) or bortezomib (1.3 mg/m^2^ intravenously once every 2 weeks; group assigned to bortezomib-based induction treatment) for 2 years. RMA-normalized values from 282 newly diagnosed MM patients (pooled standard-risk and high-risk samples from GSE19784) were rank ordered according to the expression of the ERBB1_1565484_x_at probeset. PFS and OS times were evaluated for 280 MM patients with available follow-up data (data obtained from the supplementary file 41375_2012_BFleu2012127_MOESM30_ESM.xls in from [18] that enabled comparisons of OS and PFS for these MM patients with the highest expression of ERBB1/EGFR (top 40th percentile; N = 112) versus MM patients with the lowest expression of ERBB1/EGFR (bottom 40th percentile; N = 112). Out of the 112 MM patients that expressed the highest level of ERBB1/EGFR, 33 were categorized as high-risk using the SKY-92 classifier (29%), in comparison to the 112 MM patients categorized as 18 high-risk (16%) expressing the lowest level of ERBB1/EGFR. The Kaplan–Meier (KM) method was used to investigate the PFS and OS outcome data utilizing software packages survival_3.2-13, survminer_0.4.9 and survMisc_0.5.5 operated in the R environment. Statistical significance between comparison groups was determined using the Log-rank chi-squared values and *p*-values less than 0.05 were deemed significant. Graphical representations of the survival curves were visualized using graph drawing packages implemented in the R programming environment: dplyr_1.0.7, ggplot2_3.3.5 and ggthemes_4.2.4.

Multivariate analysis of the effect of EGFR/ERBB1 expression on the PFS and OS outcomes was performed using the Cox proportional hazards model to adjust for patient prognostic characteristics as variables that included the prognostic staging data according to the International Staging System (ISS), and the 92-gene SKY signature assessed risk score. We tested whether the correlation of expression level (log_2_ RMA) was a significant independent prognostic factor controlling for each as well as all of the other variables. Estimates of the life table hazard ratios (HR) were calculated using the exponentiated regression coefficient for proportional hazards model analyses implemented in R (survival_3.2-13 ran in R version 4.1.2 (1 November 2021)). Forest plots were utilized to visualize the HRs for Cox proportional hazards model (survminer_0.4.9 ran in R version 4.1.2 (1 November 2021)).

### 2.5. Processing of the Multiple Myeloma Research Foundation (MMRF)-CoMMpass RNAseq Dataset

The entire database from 33 TCGA projects has been migrated to the NCI’s Genomic Data Commons (GDC) Data Portal (https://portal.gdc.cancer.gov/, accessed on 1 October 2022). One of the TCGA projects, the Multiple Myeloma Research Foundation (MMRF) CoMMpass study, harbors clinical information for 995 cases and RNAseq data files for 787 cases and are all made available via the GDC portal. We used the MMRF-CoMMpass RNAseq dataset for validation of the microarray-based findings regarding the prognostic effect of EGFR/ERBB1 expression. The workflow for processing RNAseq datasets standardizes the sequence alignment to the same genome build (GRCh38.p0) and harmonized quantification techniques (STAR-count; detailed in https://docs.gdc.cancer.gov/Data/Bioinformatics_Pipelines/Expression_mRNA_Pipeline/, accessed on 20 September 2022) to enable meta-analysis across multiple projects.

We utilized the latest version of the RNAseq STAR-quantified data for all 787 cases (release date 29 March 2022, version 32, STAR is implemented as a standalone C++ code. STAR is free open source software distributed under GPLv3 license and can be downloaded from http://code.google.com/p/rna-star/, accessed on 20 September 2022. And analysis performed by GDC) whereby the STAR-aligned read groups using the two-pass method were employed to generate the final alignments to calculate the expression metrics, and the quality of sequence reads was assessed by best matching the alignments of the reads to the reference genome sequence using pre-alignment with FASTQC and post-alignment with Picard Tools.

Gene level RNAseq raw count data (STAR-aligned unstranded number of reads aligned per gene per sample) were downloaded from the archived MMRF CoMMpass dataset by connecting to the GDC portal: https://gdc.cancer.gov/about-gdc/contributed-genomic-data-cancer-research/foundation-medicine/multiple-myeloma-research-foundation-mmrf. (accessed on 24 September 2022) using the Bioconductor package, Genomic-DataCommons_1.18.0 (Release date 2021-08-11 with full functionality as provided by TCGAbiolinks for accessing GDC data; https://bioconductor.org/packages/release/bioc/html/GenomicDataCommons.html (accessed on 20 September 2022)) implemented in R version 4.1.2 (1 November 2021). The mRNA expression data were deposited in files appended with “ . . . .rna_seq.augmented_star_gene_counts.tsv”. Clinical data for each MM patient were also acquired by functions provided in GenomicDataCommons_1.18.0 and the case IDs were matched with RNAseq unique identifiers utilizing the metadata (“metadata.cart.2022-09-04.json”) from the GDC portal converted into R data by running the utilities rjson_0.2.21 and stringr_1.4.0. The database consisted of 766 patients of which 267, 276 and 223 patients were ISS Stage I, Stage II and Stage III respectively. Clinical OS and prognostic staging data according to the ISS was available for 716 of these 766 patients (Stage I (N = 257), Stage II (N = 254) and Stage III (N = 185). ISS stage was not known for 20 patients.

We compared the mRNA expression for EGFR/ERBB1 using the DESeq2 package (DESeq2_1.34.0) in Stage I, Stage II and Stage III MM patients obtained from http://www.bioconductor.org/packages/release/bioc/html/DESeq2.html (accessed on 20 July 2022) implemented using R version 4.1.2 (1 November 2021) [27]. For each gene, DESeq2 uses a generalized linear model that fits read counts to negative binomial distribution to calculate the mean and variance estimates, whereby the mean is taken as a quantity proportional to the concentration of cDNA fragments from the gene in the sample and scaled by a normalization factor across all samples. The normalization method in the DESeq2 algorithm determines the counts divided by sample-specific size factors calculated from the median ratio of gene counts relative to the geometric mean per gene across all samples that accounts for sequencing depth and RNA composition for each gene. This method allows for fold-change comparisons across treatment groups in the GLM model [28-30]. Statistical significance was assessed by testing the null hypothesis that there is no differential expression across the two sample groups (Log_2_ fold change = 0) using the Wald test [27] reporting the test statistic and *p*-value for each gene. Genes were considered differentially expressed if the *p*-values were less than 0.05 after adjusting for multiple comparisons [24]. To visualize the gene expression levels in boxplots, the normalized log_2_ values were calculated from the RNAseq count data using the variance stabilization method supplied by the algorithms in vsn_3.62.0 [31]. Low count values tend to generate large fold-changes. Therefore, to calculate a more accurate log_2_ fold-change estimate, we applied a shrinkage of the log fold- change estimates toward zero when the read counts were low and variable (“normal” function in the DESeq2 package”) [32].

### 2.6. Overall Survival Analysis and Cox Proportional Hazards Model of the MMRF CoMMpass Study

The Kaplan–Meier (KM) method, log-rank chi-square test, was used to investigate the PFS and OS outcomes of MM patients utilizing the software packages survival_3.2-13, survminer_0.4.9 and survMisc_0.5.5 operated in the R environment. Statistical significance between comparison groups was determined using the log-rank chi-squared values and *p*-values less than 0.05 were deemed significant. Graphical representations of the survival curves were visualized using graph-drawing packages implemented in the R programming environment: dplyr_1.0.7, ggplot2_3.3.5 and ggthemes_4.2.4. OS curves were compared for patients expressing high levels of EGFR/ERBB1 (top 50th percentile) versus low levels of EGFR/ERBB1 (bottom 50th percentile). PFS data and biochemical measurements (beta 2 microglobulin and albumin levels) were obtained from the web repository, doi.org/10.6084/m9.figshare.11941494 [33]. The percentiles of patients expressing EGFR/ERBB1 were determined using the metric fragments per kilobase of transcript per million mapped reads (FPKM) calculation normalized to the upper quartile FPKM (FPKM-UQ) for the whole geneset in each sample.

Multivariate analysis of the effect of MMRF CoMMpass cohort groups on the PFS and OS outcomes was performed using the multivariate Cox proportional hazards model to adjust for patient prognostic characteristics. The model included (i) the expression level (high versus low FPKM-UQ values) of the EGFR/ERBB1 gene, (ii) the prognostic staging data according to the International Staging System (ISS), (iii) age, (iv) gender, (v) serum Beta 2 microglobulin levels and (vi) serum albumin levels. The multivariate Cox proportional hazards model was compared to univariate models for each of the variables to assess the independent effect of high levels of EGFR/ERBB1 expression on the PFS and OS outcomes in MM patients. Estimates of the life table hazard ratios (HRs) were calculated using the exponentiated regression coefficient for Cox proportional hazards analyses implemented in R (survival_3.2-13 ran in R version 4.1.2. Forest Plots were utilized to visualize the Hazard ratios for Cox proportional hazards model (survminer_0.4.9 ran in R version 4.1.2 (1 November 2021)).

## 3. Results

### 3.1. Amplified Expression of Selective Tyrosine Kinase Genes in Malignant Plasma Cells from MM Patients

**Figure 1.**
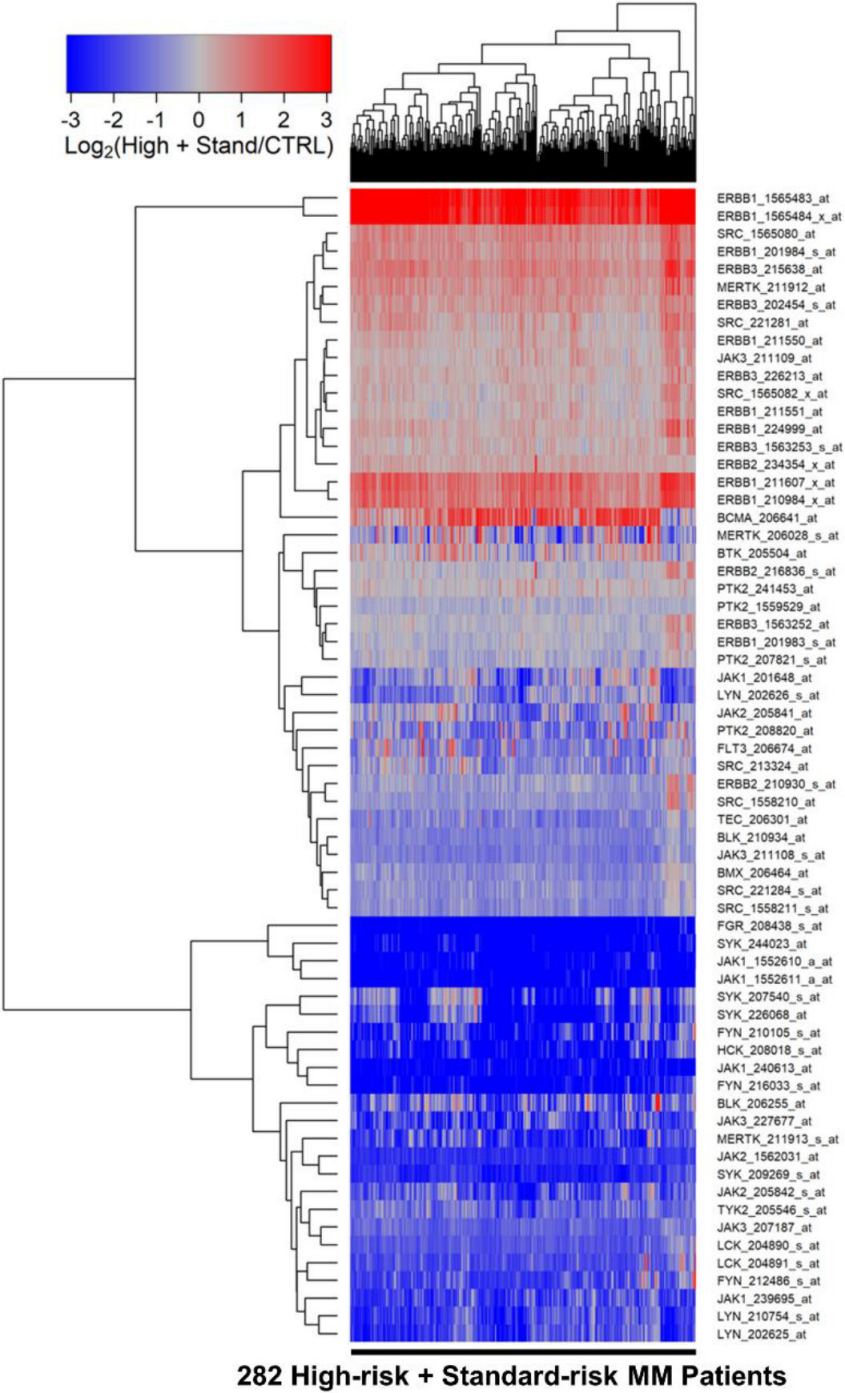
Gene Expression Levels for Tyrosine Kinases in Malignant Plasma Cells from MM Patients vs. Normal Plasma Cells from Healthy Subjects. We examined the gene expression data in the archived MM dataset GSE19784 and the control plasma cell dataset from GSE171739. The cluster figure displays the expression levels in malignant plasma cells from MM patients (high-risk and standard-risk MM patients combined) mean centered to the expression levels in normal plasma cells for log_2_-transformed fold-change values (blue represents underexpression and red color represents overexpression in MM cells). The expression levels of co-regulated probesets for both probesets (rows) and patients (columns) are organized in the depicted dendrograms. The comparison of the log_2_-transformed RMA values for normal plasma cells from 7 control samples with RMA values for malignant plasma cells from 282 MM patients showed that 46 probesets were significantly dysregulated of which 10 were upregulated in the malignant plasma cells from MM patients (FDR = 0.07). ERBB1_1565483_at was the most significantly upregulated probeset (Fold Change = 16.86; *p*-value < 10^−8^) followed by ERBB1_1565484_x_at (Fold Change = 9.91; *p*-value < 10^−8^) and ERBB1_211607_x_at (Fold Change = 2.54; *p*-value = 2.7 × 10^−6^) (Table S1). Five probesets for ERBB1 and 2 probesets for ERBB3 were significantly over expressed in MM cells. SRC (2 probesets), ERBB1, ERBB3 (2 probesets) and MERTK formed a cassette of upregulated probesets in these pooled MM patients. BCMA was co-regulated with 2 probesets for ERBB1.

We first compared the transcriptomes of purified CD138^+^ malignant plasma cells from 282 newly diagnosed MM patients with the transcriptomes of CD138^+^ normal plasma cells from 7 healthy volunteers. Epidermal growth factor receptor (EGFR) ERB1, ERBB3, SRC, and MERTK were significantly overexpressed in MM cells in comparison to normal control plasma cells (Figure 1, Table S1). ERBB1_1565483_at was the most significantly upregulated probeset (Fold Change = 16.86; *p*-value < 1 × 10^−8^) followed by ERBB1_1565484_x_at (Fold Change = 9.91; *p*-value < 1 × 10^−8^) and ERBB1_211607_x_at (Fold Change = 2.54; *p*-value = 2.7 × 10^−6^) (Table S1). It is noteworthy that the absolute EGFR/ERRB1 gene expression levels from all the MM samples regardless of the risk category or ISS stage were above the lowest expressed transcripts on the Affymetrix Human Genome U133 Plus 2.0 platform for both probesets (Figures S1 and S2, Tables S2 and S3).

Five probesets for ERBB1/EGFR and 2 probesets for ERBB3 were significantly overexpressed in MM cells. SRC (2 probesets), ERBB1/EGFR, ERBB3 (2 probesets) and MERTK formed a cassette of upregulated probesets in these pooled MM patients. The gene for the MM surface antigen BCMA, selected as a control marker gene for MM, was expressed at a 2.4-fold higher level in malignant plasma cells from MM patients than normal plasma cells (Figure 1, Table S1). BCMA was co-regulated with 2 probesets for ERBB1/EGFR.

**Figure 2.**
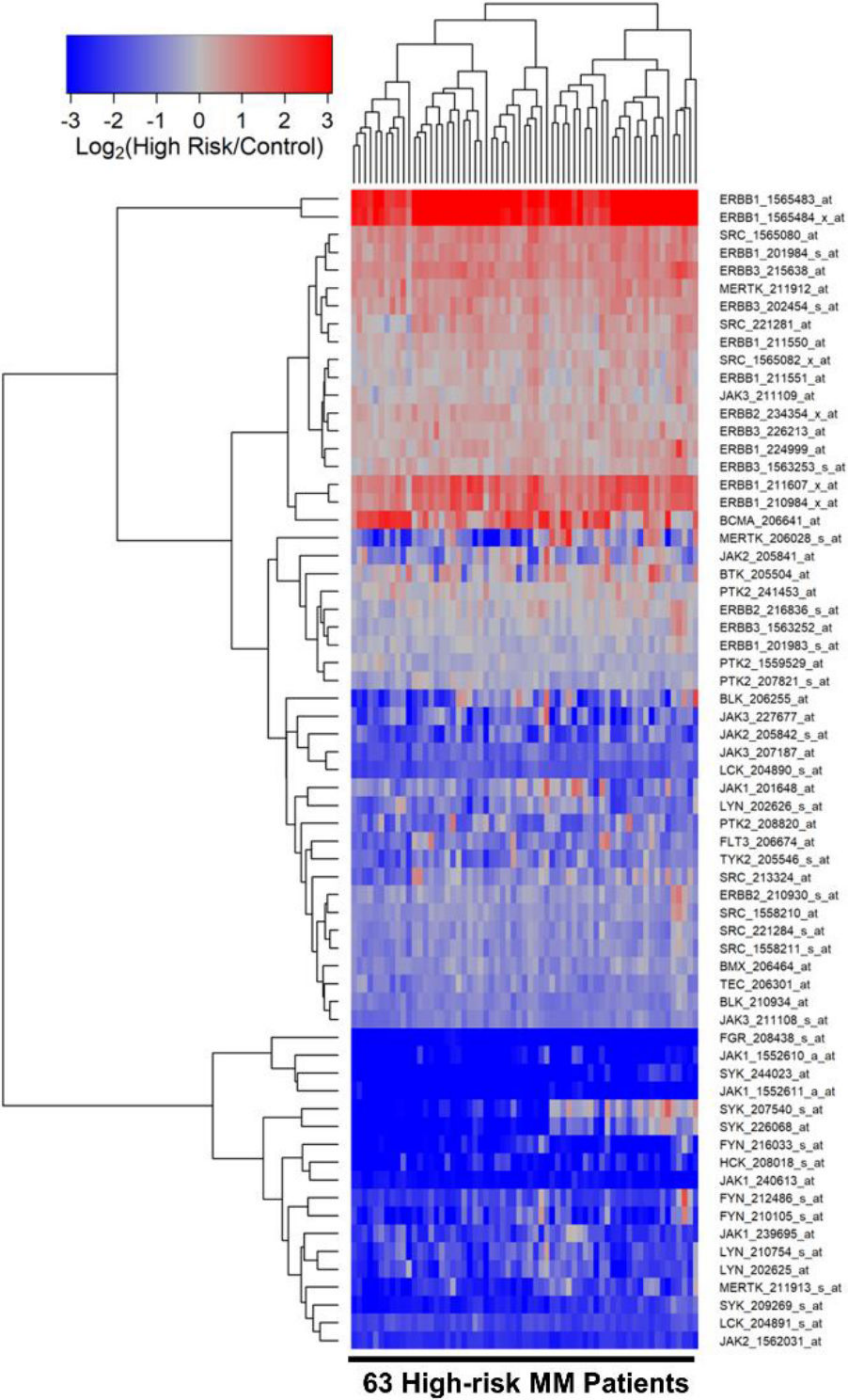
Gene Expression Levels for Tyrosine Kinases in Malignant Plasma Cells from High-Risk MM Patients vs. Normal Plasma Cells from Healthy Subjects. We examined the gene expression data in the archived MM dataset GSE19784 and the control plasma cell dataset from GSE171739. The cluster figure displays the expression levels in malignant plasma cells from 63 high-risk MM patients mean centered to the expression levels in normal plasma cells for log_2_-transformed fold-change values (blue represents underexpression and red color represents overexpression in MM cells). Patients were risk-categorized based on the patient-specific EMC-92/SKY-92 gene expression signature values. The expression levels of co-regulated probesets for both probesets (rows) and patients (columns) are organized in the depicted dendrograms. The comparison of the log_2_-transformed RMA values for normal plasma cells from 7 control samples with RMA values for malignant plasma cells from 63 high-risk MM patients showed that 47 probesets were significantly dysregulated of which 10 were upregulated in the malignant plasma cells from high-risk MM patients (FDR = 0.07). ERBB1_1565483_at was the most significantly upregulated probeset (Fold Change = 22.39; *p*-value < 1 × 10^−8^) followed by ERBB1_1565484_x_at (Fold Change = 11.94; *p*-value < 1 × 10^−8^) and ERBB1_211607_x_at (Fold Change = 2.55; *p*-value = 1.9 × 10^−6^) (Table S4). Five out of the 10 upregulated probesets were for the ERBB1 gene and 2 probesets for the ERBB3 gene. In addition, MERTK, SRC and BCMA were also significantly upregulated in malignant plasma cells. Co-regulation of BCMA and 2 probesets of ERBB1 are depicted in the cluster figure.

We next compared the transcriptomes of purified CD138^+^ malignant plasma cells from the high-risk subset of MM patients (N = 63) with the transcriptomes of CD138^+^ normal plasma cells in control samples (N = 7). 10 probesets were upregulated in malignant plasma cells from high-risk MM patients. ERBB1_1565483_at was the most significantly upregulated probeset (Fold Change = 22.39; *p*-value < 1 × 10^−8^) followed by ERBB1_1565484_x_at (Fold Change = 11.94; *p*-value < 10^−8^) and ERBB1_211607_x_at (Fold Change = 2.55; *p*-value = 1.9 × 10^−6^) (Figure 2, Table S4). Five out of the 10 upregulated probesets were for the ERBB1/EGFR gene and 2 probesets were for the ERBB3 gene. In addition, MERTK, SRC and BCMA were also significantly upregulated and BCMA was coregulated with 2 probesets of ERBB1/EGFR.

### 3.2. Differentially Amplified Expression of ERBB1/EGFR and JAK3 Genes in Malignant Plasma Cells from High-Risk MM Patients

**Figure 3.**
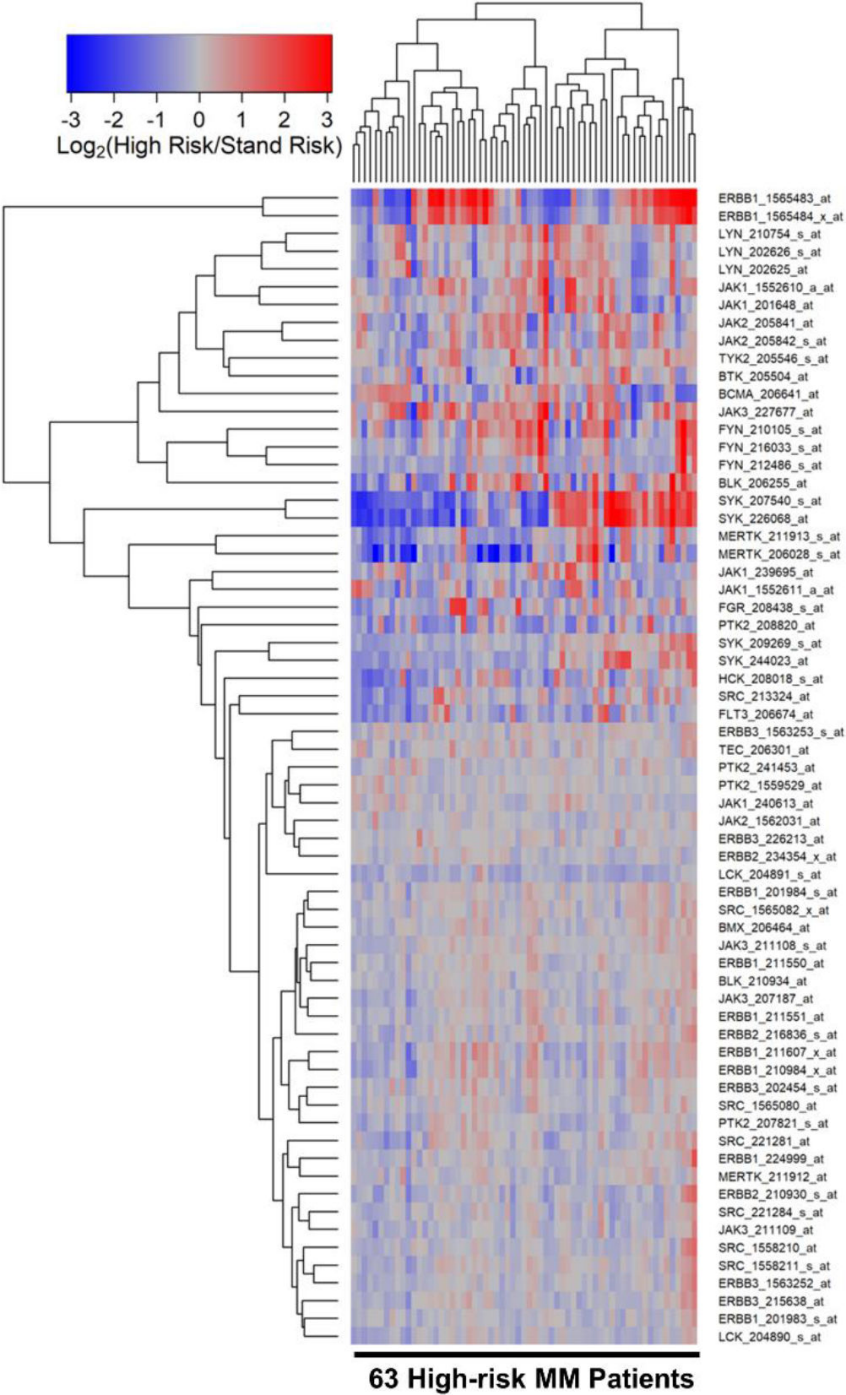
Gene Expression Levels for Tyrosine Kinases in Malignant Plasma Cells from High-Risk vs. Standard-Risk MM Patients. We examined the gene expression data in the archived MM dataset GSE19784. Patients were risk-categorized based on the patient-specific EMC-92/SKY-92 gene expression signature values. The cluster figure displays the expression levels in malignant plasma cells from high-risk MM patients mean centered to the expression levels in malignant plasma cells from standard-risk MM patients for log_2_-transformed fold change values (blue represents underexpression and red color represents overexpression in high-risk MM patients). The expression levels of co-regulated probesets for both probesets (rows) and patients (columns) are organized in the depicted dendrograms. The comparison of the log_2_-transformed RMA values for 219 standard-risk MM patients with the log_2_-transformed RMA values for 63 high-risk MM patients showed 11 dysregulated probesets of which 5 were upregulated in the high-risk subset. JAK3_227677_at (Fold Change = 1.48; *p*-value = 1.4 × 10^−7^), ERBB1_1565483_at (Fold Change = 1.44; *p*-value = 9 × 10^−7^) and ERBB1_1565484_x_at (Fold Change = 1.27; *p*-value = 1.2 × 10^−3^) were the most significantly upregulated probesets. Significant *p*-values were defined as *p* < 0.01 for FDR < 0.01 (Table S5).

We compared the transcript-level PTK expression profiles of malignant plasma cells from high-risk (N = 63) vs. standard-risk (N = 219) MM patients. Notably, the expression levels of ERBB1/EGFR and JAK3 were differentially amplified in malignant plasma cells from high-risk MM patients (Figure 3, Table S5).

### 3.3. Overexpression of EGFR/ERBB1 in MM Cells Is Associated with Augmented Expression of Transcription Factors Binding to Multiple ERBB1 Gene Promoter Sites

**Figure 4.**
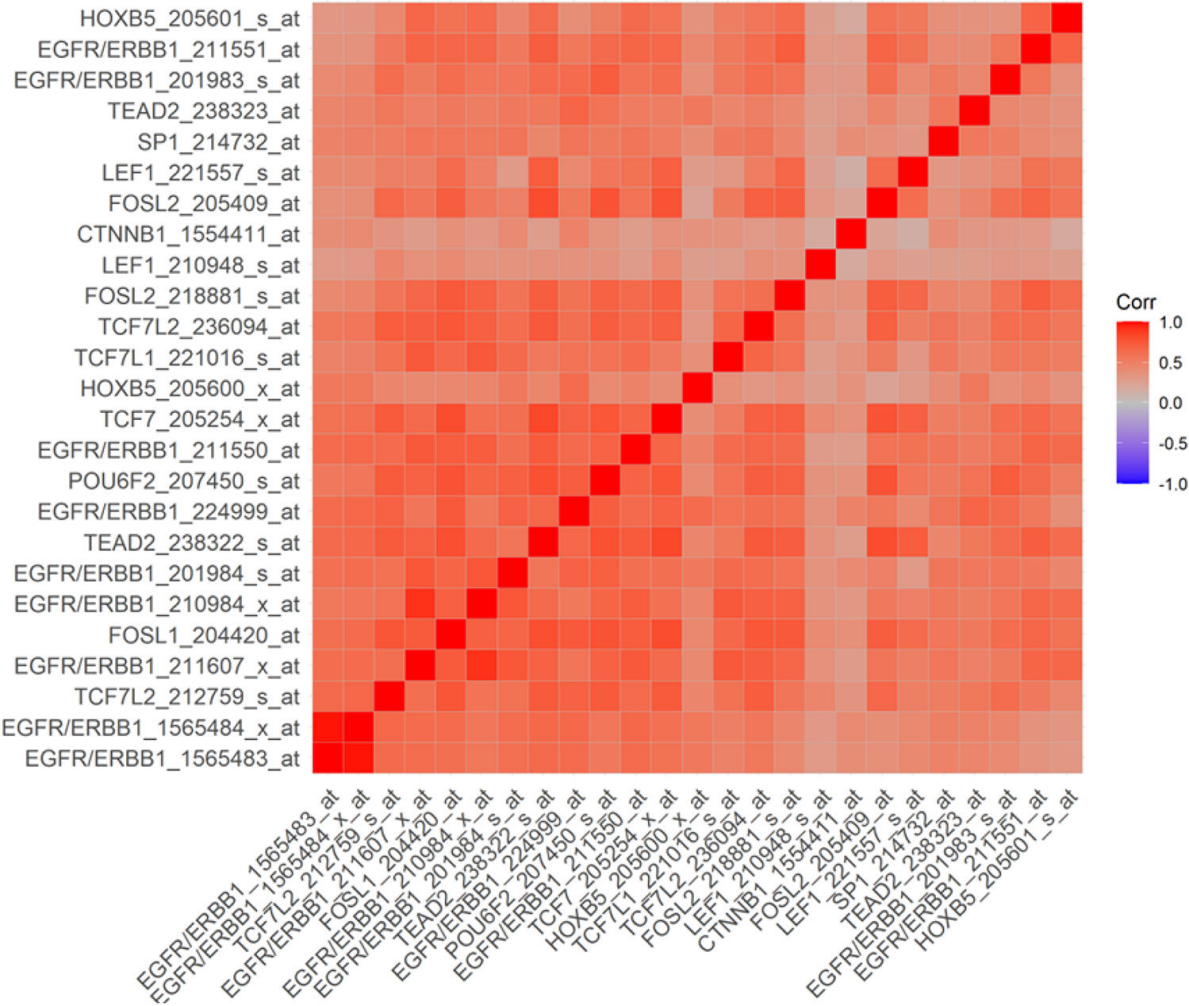
Correlation matrix of EGFR/ERBB1 probesets and transcription factors with the most significant positive correlations in MM patients.

The transcription of the EGFR/ERBB1 gene is positively regulated by several DNA binding proteins/transcription factors, including ETF [34-36], SP-1 [37-41], TCF [42,43], HOXB5 [44,45], RPF-1 [46] and AP-1 [33,47-49]. In an effort to gain insights into the mechanism of the observed transcript-level upregulation of the EGFR/ERB1 gene in MM samples, we set out to correlate the mRNA levels for EGFR/ERBB1 with the mRNA levels for DNA binding proteins/transcription factors capable of enhancing EGFR/ERBB1 expression. A total of 3600 pairwise correlations were formed of which 2302 were deemed to be significant (*p* < 0.05 and FDR < 0.1) (Figure S3). Several DNA-binding proteins with known upregulating activity on EGFR/ERBB1 gene expression exhibited transcript-level overexpression and a positive correlation with EGFR/ERBB1 expression for both EGFR/ERBB1 probesets (Figure S3, Tables S6 and S7). In particular, upregulated expression along with positive correlation with EGFR/ERB1 expression was found for (i) 3 probesets for mRNA coding TEAD2 proteins that recognize the ETF-binding site, (ii) 6 probesets (2 for JUND, 3 for FOSL2, 1 for FOSL1) for mRNA coding the DNA binding proteins that recognize the AP-1 binding site, (iii) 9 probesets for mRNA coding the DNA binding proteins that recognize the beta-Catenin TCF binding site (TCF7, TCF7L1, 4 probesets for TCF7L2 and CTNNB1, 2 probesets for LEF1), (iv) 2 probesets for mRNA coding for HOXB5, and (v) 2 probesets for mRNA coding for proteins that recognize the SP-1 binding site, and the POU6F2 that binds to the RPF-1 promoter site (Figure S3). Probesets that represented transcription factor proteins that bound to the SP-1 promoter (SP1), ETF promoter (TEAD2, 2 probesets), HOXB5 promoter (HOXB5, 2 probesets), TCF promoter (CTNNB1 and LEF1, 2 probesets), TCF7L2(TCF7L1 and TCF7, 2 probesets), RPF-1 promoter (POU6F2) and AP-1 promoter (FOSL1 and FOSL2, 2 probesets) were amongst the most significantly positively correlated probesets (Figure 4).

Pairwise Pearson correlations were performed between EGFR/ERBB1 and transcription factor probesets (60 probesets representing 21 genes, see Figure S3) and the correlation coefficients were calculated across 282 MM patients (GSE19784) (Corr). Depicted in Figure 4 are the most significant positively correlated probesets (25 probesets wit *p* < 0.001, FDR = 0.006) are depicted on the heatmap ranging from positive correlations (red) to negative correlations (blue) organized according to similarly expressed probesets. All 9 probesets for EGFR/ERRB1 were positively correlated with each other. Probesets that represented transcription factor proteins that bound to the SP-1 promoter (SP1), ETF promoter (TEAD2, 2 probesets), HOXB5 promoter (HOXB5, 2 probesets), TCF promoter (CTNNB1 and LEF1, 2 probesets), TCF7L2 (TCF7L1 and TCF7, 2 probesets), RPF-1 promoter (POU6F2) and AP-1 promoter (FOSL1, FOSL2, 2 probesets) were amongst the most significantly positively correlated probesets.

### 3.4. Amplified Expression of ERBB1/EGFR Is Associated with Poor PFS and OS in Newly Diagnosed MM

We next evaluated the potential impact of ERBB1/EGFR expression levels on the PFS and OS of newly diagnosed MM patients by comparing the PFS and OS times for MM patients with the highest expression level of ERBB1/EGFR (i.e., top 40% with the highest observed expression level; N = 112) with the PFS and OS times for MM patients with the lowest expression level of ERBB1/EGFR (i.e., bottom 40% with the lowest observed expression level; N = 112). Notably, patients with the highest ERBB1/EGFR expression level had a significantly shorter PFS than patients with the lowest ERBB1/EGFR expression level (Figure S4, Panel A). There were 82 events recorded for PFS in patients with the highest level of expression for ERBB1/EGFR (Median PFS = 20.6 months; 95% CI 17.9–27.5), compared to 64 events that were recorded for patients with the lowest level of expression for ERBB1/EGFR (Median PFS = 34.7 months; 95% CI 28.4–40.6) (*p*-value: 0.0011). Likewise, the OS outcome was significantly worse for the patient subset with the highest ERBB1/EGFR expression level (Figure S4, Panel B). There were 47 events recorded for OS in patients with the highest level of expression for ERBB1/EGFR (Median OS = 53.7 months compared to 28 events that were recorded for patients with the lowest level of expression for ERBB1/EGFR (Median OS not reached) (*p*-value: 0.006).

This effect of ERBB1/EGFR expression on the PFS and OS outcomes may not be independent of other prognostic markers: Among the 112 patients with the highest expression level of ERBB1/EGFR, we observed a significantly higher proportion of high-risk patients based on the SKY-92 classification than among the 112 patients with the lowest expression level of ERBB1/EGFR (33/112 = 29% vs. 18/112 = 16%, *p*-value = 0.025).

We next examined the effects of ERBB1/EGFR expression on PFS and OS in univariate and multivariate Cox proportional hazards models that also included SKY-92 gene signature-based risk assignment and prognostic ISS stage as categorical covariates. High EGFR/ERBB1 expression was associated with significantly increased hazard ratios (HR) for unfavorable PFS outcomes in both univariate (HR = 1.85 (1.3–2.62)) and multivariate (HR = 1.5 (1.05–2.15)) (Figure S5A,B). Cox proportional hazards models. High EGFR/ERBB1 expression was also associated with significantly increased HRs for unfavorable OS outcomes in both univariate (HR = 2.28 (1.38–3.78)) and multivariate (HR =1.75 (1.05–2.92)) Cox proportional hazards models (Figure S5C,D). The impact of high EGFR/ERBB1 expression on the PFS and OS outcomes remained significant even after accounting for the prognostic effects of the risk category and ISS stage (Supplementary Figure S5A-D).

### 3.5. Analyses Using the MMRF-CoMMpass RNAseq Dataset for Validation of the Microarray-Based Findings from the HOVON65/GMMG-HD4 Trial Regarding the Prognostic Effect of EGFR/ERBB1 Expression

The randomized HOVON65/GMMG-HD4 Phase 3 study evaluating the clinical efficacy of bortezomib induction/maintenance versus classic cytotoxic drugs and thalidomide maintenance relied in part on cytotoxic agents such as doxorubicin and vincristine that are no longer routinely used as part of frontline induction therapy in MM patients [50]. To ensure that EGFR/ERBB1 expression remains relevant with modern induction regimens, we next used the MMRF-CoMMpass RNAseq dataset generated in patients treated with contemporary backbone combinations of immunomodulatory drugs (IMiDs), proteosome inhibitors, and dexamethasone for validation of the microarray-based findings from the HOVON65/GMMG-HD4 study regarding the prognostic effect of EGFR/ERBB1 expression [51-53]. We first determined the lowest detection level for the normalized, variance stabilized log_2_ count value at which there were zero alignments to the EGFR/ERBB1 gene in 766 MM patients with ISS staging information. Only cells from 157 patients (20.5%) across all 3 prognostic ISS stages had zero alignments to the EGFR/ERBB1 gene (Figure S6).

**Figure 5.**
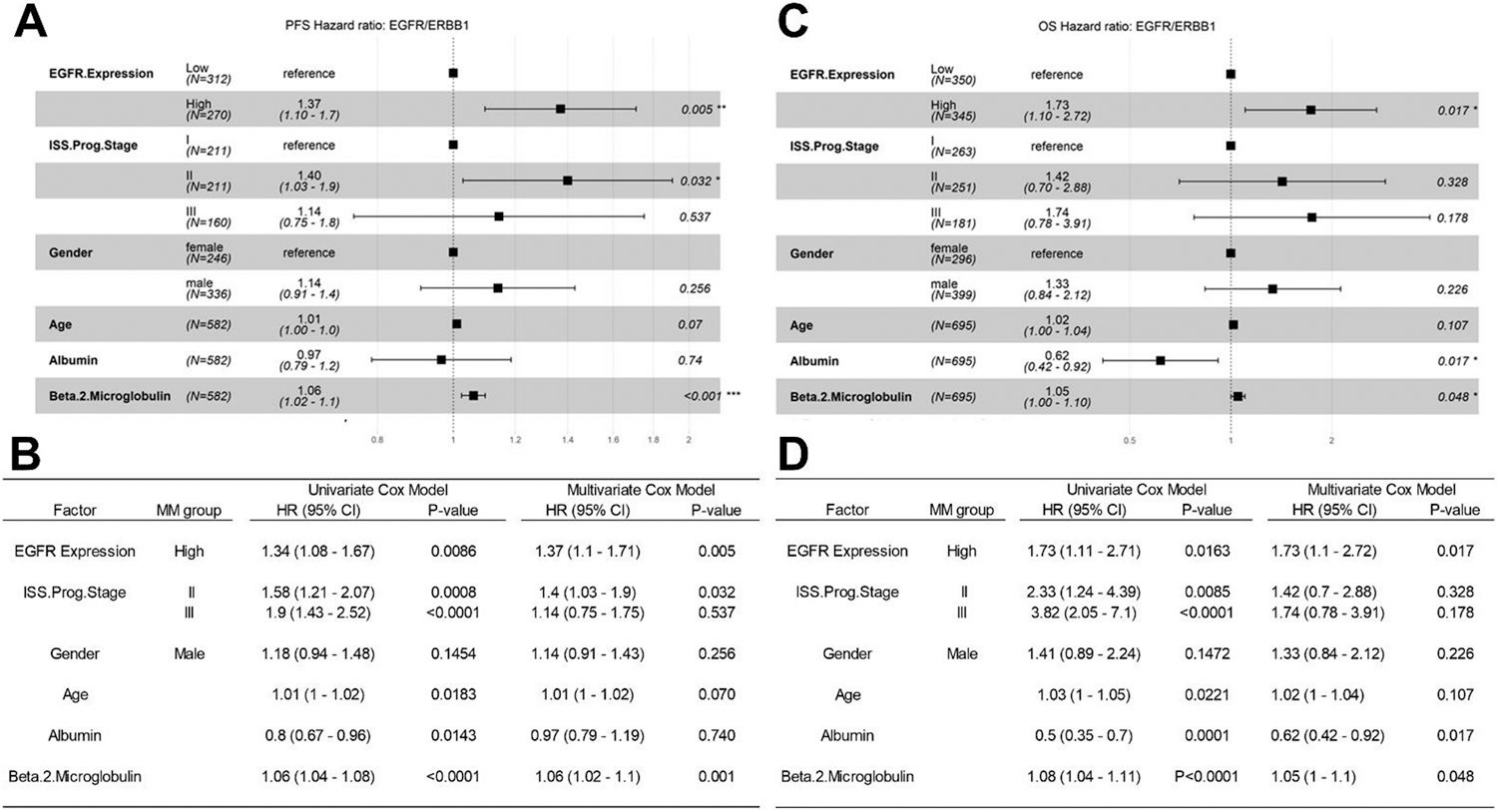
The unfavorable impact of high EGFR/ERBB1 expression on PFS and OS outcomes of MM patients from the MMRF-CoMMpass study in univariate and multivariate Cox proportional hazards models. (**A,B**) Comparison of the PFS times for 582 evaluable patients showed a significant increase in HR for patients with high expres-sion of EGFR/ERBB1 compared to patients with low EGFR/ERBB1 expression in the multivariate model. (**C,D**) OS relation-ships were evaluable for 695 patients). Significant *p*-values in B and D are indicated by *, ** and *** for *p* < 0.05, *p* < 0.01 and *p* < 0.001 respectively.

The Kaplan–Meier (KM) method, log-rank chi-square test, was then used to investigate the PFS (N = 598) and OS (N = 716) of evaluable MM patients in relationship to their RNAseq-based EGFR/ERBB1 expression levels. Patients with higher levels of EGFR/ERBB1 expression had worse outcomes with significantly shorter PFS and OS times (Figure S7). We next examined the effects of ERBB1/EGFR expression on PFS and OS in univariate and multivariate Cox proportional hazards models. Higher EGFR/ERBB1 expression was associated with significantly increased hazard ratios (HR) for unfavorable PFS outcomes in both univariate (HR = 1.85 [1.3–2.62]) and multivariate (HR = 1.5 [1.05–2.15]) (Figure 5A) Cox proportional hazards models. Higher EGFR/ERBB1 expression was also associated with significantly increased HRs for unfavorable OS outcomes in both univariate (HR = 2.28 (1.38–3.78)) and multivariate (HR =1.75 (1.05–2.92)) Cox proportional hazards models (Figure 5B). The impact of higher EGFR/ERBB1 expression on the PFS (HR: 1.37, *p* = 0.005) and OS (HR: 1.73, *p* = 0.017) outcomes remained statistically significant even after accounting for the prognostic effects of the prognostic ISS stage, age, gender, serum albumin level and serum beta 2 microglobulin level (Figure 5).

Gene level normalized expression values (FPKM-UQ) obtained from GDC data portal aligned to the EGFR/ERBB1, sequences (GRCh38.p0 genome build)) was correlated with PFS (Figure 5 Panels A, B) and OS (Figure 5 Panels C, D) times using the univariate and multivariate Cox proportional hazards models. We investigated 3 categorical variables: (i) EGFR expression (i.e., High EGFR/ERBB1 expression, top 50% of patients versus Low EGFR/ERBB1 expression, Bottom 50% of patients), (ii) ISS prognostic stage (ISS.Prog.Stage) and (iii) Gender, and 3 linear co-variates (Age, serum albumin levels and serum beta 2 microglobulin levels). (A, B) Comparison of the PFS times for 582 evaluable patients showed a significant increase in HR for patients with high expression of EGFR/ERBB1 compared to patients with low EGFR/ERBB1 expression in the multivariate model. The comparison of the multivariate model with the univariate testing of each of these variables showed that the increased HR observed in patients with high levels of EGFR/ERBB1 was similar in both models: Univariate model HR = 1.34 (1.08–1.67) versus multivariate model HR = 1.37 (1.1–1.71). (C, D) OS relationships were evaluable for 695 patients). The multivariate model exhibited an increase in HR for patients expressing high levels of EGFR/ERBB1 (HR = 1.73 (1.1–2.72) that was virtually identical to the HR of 1.73 (1.1–2.71) in the univariate model (D). Depicted are the Forest plots along with the corresponding HRs and *p*-values for each covariate. The tables compare the effect of each variable considering univariate and multivariate application of the Cox proportional hazards model. Higher levels of EGFR/ERBB1 expression have an unfavorable impact on both PFS and OS in MM patients.

## 4. Discussion

Our detailed comparative analysis of the transcript-level PTK expression profiles of purified CD138^+^ malignant plasma cells from newly diagnosed MM patients vs. normal plasma cells showed that ERBB1/EGFR, ERBB3, SRC, and MERTK were significantly overexpressed in MM cells in comparison to normal control plasma cells. Further, ERBB1/EGFR and JAK3 exhibited differentially amplified expression in malignant plasma cells from high-risk MM patients. These results provide new insights regarding the clinical impact potential of targeting ERBB1/EGFR in high-risk MM.

Notably, amplified expression of ERBB1/EGFR emerged as a prognostic biomarker in both the dataset from the HOVON65/GMMG-HD4 study (depicted in Figure S4) as well as the MMRF-CoMMpass RNAseq validation dataset (depicted in Figure S7): Patients with the highest ERBB1/EGFR expression levels had significantly worse PFS and OS outcomes than patients with lowest ERBB1/EGFR expression levels. Hence, this finding was not an artifact unique to a specific clinical study. Furthermore, the impact of high EGFR/ERBB1 expression on the PFS and OS outcomes remained statistically significant even after accounting for the effects of the SKY-92 gene signature-based risk assignment, prognostic ISS stage, age, gender, serum albumin level and serum beta 2 microglobulin level in multivariate Cox proportional hazards models shown in Figures S5 and 5.

ERBB1/EGFR overexpression is associated with rapid invasive growth, progression, and metastasis of solid tumors [54-60]. Furthermore, ERBB1/EGFR expression has been associated with resistance to immune checkpoint inhibitors (ICI) in lung cancer [61]. Small molecule TKI directed to the kinase domain of ERBB1/EGFR, such as afatinib, erlotinib, gefitinib, and osimertinib (Table 1), inhibit the kinase activity and abrogate the biochemical signals and downstream biological effects associated with the PTK activity of ERBB1/EGFR [56-58]. Likewise, monoclonal antibodies directed against the extracellular domain of ERBB1/EGFR, such as cetuximab block the binding of EGF and other ligands, such as amphiregulin (AREG), to the ERBB1/EGFR receptor and inhibit the ERBB1/EGFR signaling pathway that contributes to the survival and proliferation of EGFR-positive malignant cells [57,60]. ERBB1/EGFR-targeting monoclonal antibodies such as cetuximab (CTX), panitumumab, nimotuzumab, necitumumab have been incorporated into contemporary treatment programs for ERBB1/EGFR^+^ advanced solid tumors [54-60]. Targeted nanomedicines and antibody-drug conjugates have also been developed for ERBB1/EGFR-directed treatment of several cancer types [57]. The experience and knowledge gained in the clinical development of ERBB1/EGFR-targeting FDA-approved therapeutics as precision medicines against other human cancer types should facilitate their development as potential precision medicines against MM as well.

Tumors with activating ERBB1/EGFR exon 19 deletions and exon 21 mutations have exhibited favorable responses to TKI of ERBB1/EGFR [58]. Although the first generation TKI of ERBB1/EGFR have not shown meaningful clinical activity in cancer patients with wildtype ERBB1/EGFR or mutant ERBB1/EGFR with T790M, new-generation TKI such as Afatinib are active even against wildtype or T790M mutant ERBB1/EGFR [56]. It is noteworthy that tumors with ERBB1/EGFR exon 20 insertion mutations are significantly less sensitive to both TKI and monoclonal antibodies targeting ERBB1/EGFR [62]. Clinical evaluation of ERBB1/EGFR targeting therapeutics in MM patients would benefit from integrated biomarker research to determine if MM cells display or acquire any ERBB1/EGFR mutations that may affect treatment outcomes. TKI of ERBB1/EGFR cause treatment-emergent side effects some of can be associated with potentially fatal serious toxicities such as lung injury/interstitial lung disease, especially in patients with preexisting pulmonary disease [63,64]. Careful monitoring and risk mitigation strategies will be required to maximize patient safety in clinical trials of ERBB1/EGFR inhibitors in MM patients.

Our results extend previous work that has implicated the EGF-family growth factor AREG as an autocrine growth factor that binds selectively to ERBB1/EGFR on MM cells and promotes their growth and survival [65]. Further, the ERBB1/EGFR ligand EGF derived from bone marrow stromal cells has been shown to stimulate the clonogenic growth of MM cells [66]. The ERBB1/EGFR inhibitor Iressa induced apoptosis in primary MM cells and enhanced the apoptotic activity of dexamethasone [65]. RAS or RAF mutations may render MM cells resistant to treatment with ERBB1/EGFR1 inhibitors and limit any potential clinical benefit to MM patients with a KRAS/NRAS/BRAF triple wildtype genotype [67]. MM-derived exosomes have been shown to contain AREG and activate the EGF signaling pathway in the bone microenvironment leading to osteoclastogenesis [68]. As AREG selectively binds to ERBB1/EGFR, TKI of ERBB1/EGFR could also inhibit the progression of osteolytic bone disease in MM. It is noteworthy that the anti-EGFR antibody Cetuximab was evaluated in a pilot study as a single agent in patients with refractory or relapsed MM who had previously received at least one line of prior treatment and were not eligible to undergo autologous stem cell transplantation [69]. VonTreschkow et al. reported an overall response rate of 27% with acceptable tolerability [69].

**Figure 6.**
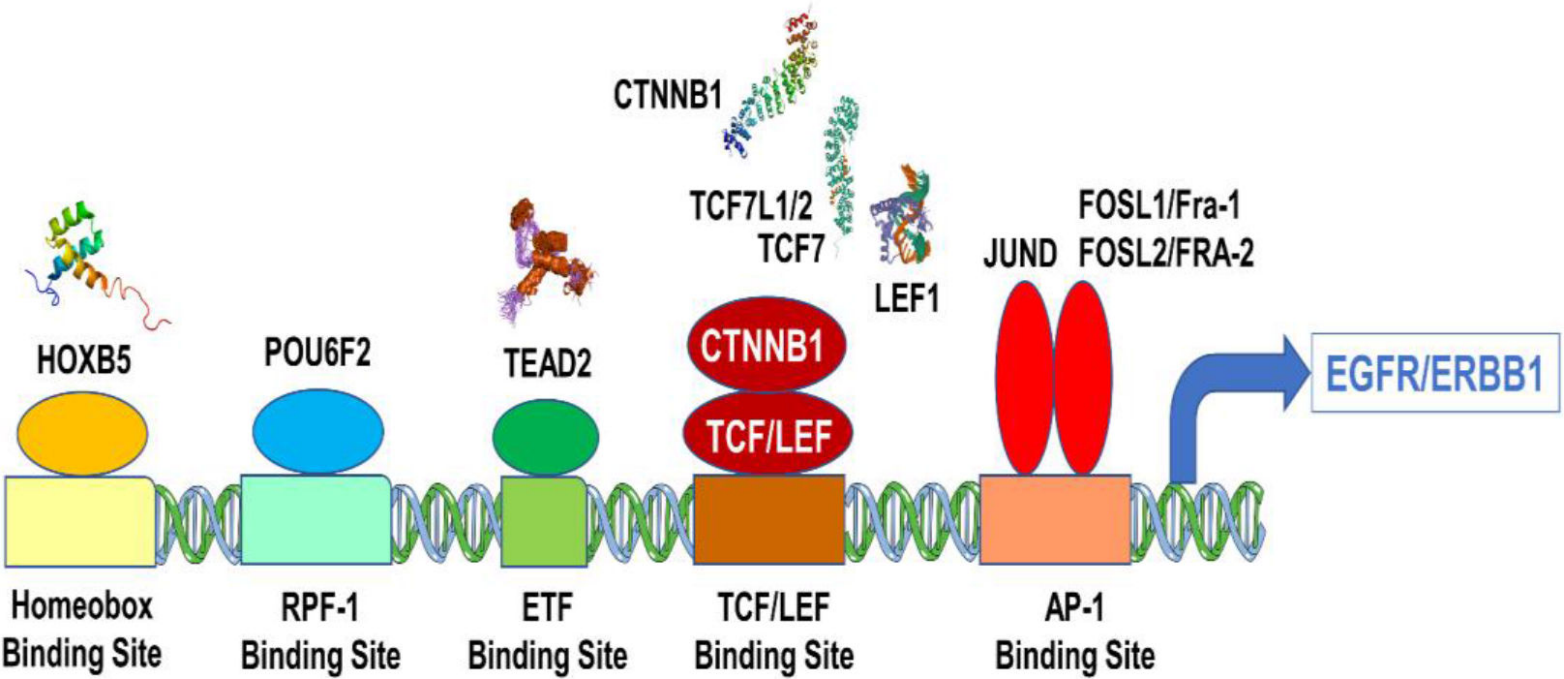
Transcription factor genes that are positively correlated with EGFR/ERRB1 expression in Multiple Myeloma (MM) patients.

We hypothesized that the augmented expression of the EGFR/ERBB1 in MM cells could be owing to the amplified expression of transcription factors that are known to upregulate EGFR/ERBB1 expression. We therefore examined if the ERBB1/EGFR expression in MM cells is correlated with expression levels of such DNA binding proteins. Our analyses provided unprecedented evidence that the amplified expression of EGFR/ERBB1 mRNA in MM cells was positively correlated with increased expression levels of mRNAs for several DNA binding proteins/transcription factors, including ETF [34-36], SP-1 [37-41], TCF [42,43], HOXB5 [44,45], RPF-1 [46] and AP-1 [33,47-49]. Based on these findings we propose a model according to which the mechanism of the observed transcript-level upregulation of the EGFR/ERB1 gene in MM samples is related to the upregulated expression of the correlated DNA binding proteins (Figure 6). The mRNA for these transcription factors were highly positively correlated with EGFR/ERBB1 gene upregulation (*p* < 0.0001) in MM patients.

Correlation coefficients of gene expression levels calculated between EGFR/ERBB1 and transcription factor probesets (282 MM patients (GSE19784)) identified 11 genes that were significantly positively correlated with EGFR/ERBB1 expression. Depicted in Figure 6 are the mRNAs that coded for transcription factor proteins (oval shapes) bound to DNA sequence binding sites for these transcription factors (rectangles).

Attributes: By Jawahar Swaminathan and MSD staff at the European Bioinformatics Institute—http://www.ebi.ac.uk/pdbe-srv/view/images/entry/2hzd600.png, (accessed on 19 September 2022) displayed on http://www.ebi.ac.uk/pdbe-srv/view/entry/2hzd/summary, (accessed on 19 September 2022) Public Domain, https://commons.wikimedia.org/w/index.php?curid=7072395 (accessed on 19 September 2022); https://www.vector4free.com/free-vectors/dna (accessed on 19 September 2022); http://www.pdb.org/pdb/explore/explore.do?structureId=2BCT (accessed on 19 September 2022); https://www.rcsb.org/structure/1JDH (accessed on 19 September 2022); https://upload.wikimedia.org/wikipedia/commons/0/0c/Protein_HOXB5_PDB_1hom.png (accessed on 19 September 2022).

The SRC family PTK including SRC, BLK, HCK, LYN, FYN, LCK have important regulatory function related to survival, proliferation, and apoptosis of lymphoid cells [26,65-67]. FDA-approved SRC kinase inhibitors have become part of the standard of care in the treatment of Philadelphia chromosome positive (Ph^+^) acute lymphoblastic leukemia (ALL) [70-85], Table 1. In our current study, we discovered the amplified expression of the SRC PTK in MM cells. The availability of FDA-approved potent inhibitors of SRC family PTK such as ponatinib, bosutinib, and dasatinib (Table 1) provides an opportunity to evaluate their clinical impact potential for MM patients in proof-of-concept clinical studies.

Janus family protein tyrosine kinases, including JAK1, JAK2, JAK3 and TYK2, have important regulatory roles in multiple signal transduction pathways intimately linked to survival, proliferation, maturation, and function of normal as well as malignant lymphoid cells [86-88]. An intriguing new finding gained in the present study was the differential upregulation of JAK3 expression in malignant plasma cells from high-risk MM patients. JAK3 associates with the common cytokine receptor γ chain (γc) and thereby regulates signaling by the cytokines Interleukin (IL)-2, IL-4, IL-7, IL-9, IL-15, and IL-21 [89]. Tofacitinib (Xeljanz^®^) is a potent, selective JAK inhibitor that preferentially inhibits JAK1 and JAK3 used for the treatment of moderate to severe active rheumatoid arthritis (RA) (Table 1) [90-92], Tofacitinib had promising in vitro activity on MM cells [93]. Further, the expression of JAK1 and JAK2 were previously reported to be upregulated in some MM cells and the JAK1/JAK2 inhibitor Ruxolitinib in combination with Bortezomib, Itacitinib or Daratumumab inhibited JAK/STAT3 phosphorylation, inhibited in vitro and in vivo myeloma cell growth and induced cell apoptosis [94-96]. In a phase I clinical trial, Ruxolitinib exhibited promising early clinical activity in R/R MM patients [97], Contemporary induction protocols for MM employ Pis such as bortezomib and Carfilzomib [2], PIs trigger the apoptotic death of MM cells by contributing to an exaggerated unfolded protein response (UPR) pathway and ER stress [2]. They further impair the survival-promoting interactions between MM cells and stromal elements in the bone marrow microenvironment, and they promote the immunogenic death of damaged MM cells [2]. The combination of PIs with JAK inhibitors may potentiate their pro-apoptotic activity against MM cells. Likewise, JAK inhibitors could be combined with other apoptosis-inducing MM drugs, such as the BCL-2 homology 3 (BH3)-mimetic Venetoclax and inhibitors of MCL-1 such as AMG-176 and MIK665 [1]. Recently, serious concerns have emerged regarding the safety profile of JAK inhibitors, especially JAK3 inhibitor Tofacitinib as well as Baricitinib, and Upadacitinib [98]. In particular, there was an increased risk of serious heart-related events such as heart attack or stroke, cancer, blood clots, and death with arthritis and ulcerative colitis medicines Xeljanz and Xeljanz XR (tofacitinib). Therefore, targeting JAK3 in MM will require risk mitigation strategies to reduce the incidence of serious cardiovascular side effects. Several new JAK3 inhibitors have been developed and some of these new-generation inhibitors could have more favorable safety profiles [99-102]. The favorable safety profile and promising anti-tumor activity of the dual-function JAK3-ERBB1/EGFR inhibitor WHI-P131 also warrants further studies [103].

## Supplementary Material

onco-1871691-supplementary 2 Figure S1: Absolute ERBB1 Gene Expression Levels in Malignant Plasma Cells from SKY-92 Risk-assessed MM Patients and Normal CD138+ Plasma Cells from Healthy Subjects
Figure S2: Absolute ERBB1 Gene Expression Levels in Malignant Plasma Cells from SKY-92 Risk-assessed MM Patients Categorized According to Prognostic ISS Staging Criteria
Figure S3: Correlation matrix of EGFR/ERBB1 probesets and transcription factors in MM patients
Figure S4: Higher ERBB1/EGFR Expression Level is Associated with Shorter PFS and OS in Newly Diagnosed MM patients
Figure S5: The unfavorable impact of higher EGFR/ERBB1 expression level on PFS and OS outcomes of MM patients in univariate and multivariate Cox proportional hazards models
Figure S6: Expression levels for EGFR/ERBB1 in malignant plasma cells from 766 newly diagnosed MM patients according to prognostic ISS stage
Figure S7: Higher ERBB1/EGFR Expression Level is Associated with Shorter PFS and OS in Newly Diagnosed MM patients from the MMRF-COMPASS study
Table S1: Gene expression levels for tyrosine kinases in malignant plasma cells from MM patients vs. normal plasma cells from healthy subjects
Table S2: Descriptive statistics for raw ERBB1 gene expression levels in malignant plasma cells from SKY-92 risk-assessed MM patients and normal CD138+ plasma cells from healthy subjects
Table S3: Descriptive statistics for ERBB1 probesets in malignant plasma cells from newly diagnosed MM patients risk assessed using ISS staging criteria
Table S4: Gene expression levels for tyrosine kinases in malignant plasma cells from high-risk MM patients vs. normal plasma cells from healthy subjects
Table S5: Gene expression levels for tyrosine kinases in malignant plasma cells from high-risk vs. standard-risk MM patients
Table S6: Correlated expression of transcription factor genes with EGFR/ERBB1 gene expression (Probeset: EGFR_1565484_x_at) in MM patients
Table S7: Correlated expression of transcription factor genes with EGFR/ERBB1 gene expression (Probeset: EGFR_1565483_at) in MM patients.

## Figures and Tables

**Table 1.** FDA-Approved Inhibitors of ERBB1/EGFR, SRC, and JAK1-3.

Target Kinase	Drug	Brand
ABL1, SRC	Bosutinib	Bosulif, SKI-606
ABL1, SRC, CKIT	Dasatinib	BMS-354825, Sprycell
ABL1, SRC	Ponatinib	Iclusig
EGFR	Gefitinib	ZD1839, Iressa
EGFR	Dacomitinib	PF-00299804, Visimpro
EGFR	Erlotinib	OSI-744, Tarceva
EGFR T790M	Osimertinib	AZD-9292, Tagrisso
EGFR with exon 20 insertions	Mobocertinib	TAK-788, AP-32788, EXKIVITY
ErbB1/2/4	Afatinib	Gilotrif, Tovok
ErbB1/2/HER2	Lapatinib	Tykerb
JAK1	Upadacitinib	ABT-494, Rinvoq
JAK1/2	Baricitinib	Olumiant, LY 3009104
JAK1/2/3, Tyk	Ruxolitinib	Jakafi
JAK2	Fedratinib	TG101348, Inrebic
JAK3	Tofacitinib	Xeljanz

## Data Availability

Raw Affymetrix. CEL data files on gene expression profiles of MM cells analyzed in the current study were obtained from publicly available datasets deposited in the NCBI repository. The working database included data on purified normal plasma cell samples from healthy volunteers (N = 7; GSE171739), purified malignant plasma cell samples from newly diagnosed high-risk MM patients (N = 63; GSE19784) and purified malignant plasma cell samples from newly diagnosed standard-risk MM patients (N = 219; GSE19784). For the validation dataset, gene level RNAseq raw count data (STAR aligned unstranded number of reads aligned per gene per sample) were downloaded from the archived MMRF CoMMpass dataset by connecting to the GDC portal: https://gdc.cancer.gov/about-gdc/contributed-genomic-data-cancer-research/foundation-medicine/multiple-myeloma-research-foundation-mmrf. (accessed on 24 September 2022)) using the Bioconductor package, GenomicDataCommons_1.18.0 (Release date 2021-08-11 with full functionality as provided by TCGAbiolinks for accessing GDC data; https://bioconductor.org/packages/release/bioc/html/GenomicDataCommons.html (accessed on 20 September 2022)) implemented in R version 4.1.2 (1 November 2021). The mRNA expression data were deposited in files appended with “ . . . .rna_seq.augmented_star_gene_counts.tsv”. Clinical data for each MM patient was also acquired by functions provided in GenomicDataCommons_1.18.0 and the case IDs were matched with RNAseq unique identifiers utilizing the metadata (“metadata.cart.2022-09-04.json”) from the GDC portal converted into R data by running the utilities rjson_0.2.21 and stringr_1.4.0. The database consisted of 766 patients of which 267, 276 and 223 patients were ISS Stage I, Stage II and Stage III respectively.
