# Supplementary material for: ERBB1/EGFR and JAK3 Tyrosine Kinases as Potential Therapeutic Targets in High-Risk Multiple Myeloma
Source: Onco (Basel). Author manuscript; Available in PMC 2022 Dec 1. (PMC9610889; doi:10.3390/onco2040016)
Supplement: onco-1871691-supplementary 2 — Figure S1: Absolute ERBB1 Gene Expression Levels in Malignant Plasma Cells from SKY-92 Risk-assessed MM Patients and Normal CD138+ Plasma Cells from Healthy Subjects Figure S2: Absolute ERBB1 Gene Expression Levels in Malignant Plasma Cells from SKY-92 Risk-assessed MM Patients Categorized According to Prognostic ISS Staging Criteria Figure S3: Correlation matrix of EGFR/ERBB1 probesets and transcription factors in MM patients Figure S4: Higher ERBB1/EGFR Expression Level is Associated with Shorter PFS and OS in Newly Diagnosed MM patients Figure S5: The unfavorable impact of higher EGFR/ERBB1 expression level on PFS and OS outcomes of MM patients in univariate and multivariate Cox proportional hazards models Figure S6: Expression levels for EGFR/ERBB1 in malignant plasma cells from 766 newly diagnosed MM patients according to prognostic ISS stage Figure S7: Higher ERBB1/EGFR Expression Level is Associated with Shorter PFS and OS in Newly Diagnosed MM patients from the MMRF-COMPASS study Table S1: Gene expression levels for tyrosine kinases in malignant plasma cells from MM patients vs. normal plasma cells from healthy subjects Table S2: Descriptive statistics for raw ERBB1 gene expression levels in malignant plasma cells from SKY-92 risk-assessed MM patients and normal CD138+ plasma cells from healthy subjects Table S3: Descriptive statistics for ERBB1 probesets in malignant plasma cells from newly diagnosed MM patients risk assessed using ISS staging criteria Table S4: Gene expression levels for tyrosine kinases in malignant plasma cells from high-risk MM patients vs. normal plasma cells from healthy subjects Table S5: Gene expression levels for tyrosine kinases in malignant plasma cells from high-risk vs. standard-risk MM patients Table S6: Correlated expression of transcription factor genes with EGFR/ERBB1 gene expression (Probeset: EGFR_1565484_x_at) in MM patients Table S7: Correlated expression of transcription factor genes with EGFR/ERBB1 gene expression (Pr [file NIHMS1843463-supplement-onco-1871691-supplementary_2.pdf]

Supplemental Tables

Table S1. Gene expression levels for tyrosine kinases in malignant plasma cells from MM patients vs. normal plasma cells from healthy subjects.

Probeset	Fold Change (High + Standard Risk/Control)	Linear Contrast (P-value)
ERBB1_1565483_at	16.86	$<1 \times 10^{-8}$
ERBB1_1565484_x_at	9.91	$<1 \times 10^{-8}$
ERBB1_211607_x_at	2.54	2.7×10^{-6}
BCMA_206641_at	2.4	1.1×10^{-5}
ERBB1_210984_x_at	2.33	2.1×10^{-5}
ERBB3_215638_at	2.02	4.3×10^{-4}
SRC_1565080_at	1.86	1.7×10^{-3}
ERBB1_201984_s_at	1.73	5.9×10^{-3}
MERTK_211912_at	1.72	6.3×10^{-3}
ERBB3_202454_s_at	1.54	2.9×10^{-2}
SRC_221281_at	1.4	9.1×10^{-2}
ERBB1_224999_at	1.36	1.2×10^{-1}
ERBB2_234354_x_at	1.33	1.5×10^{-1}
ERBB1_211550_at	1.29	2.0×10^{-1}
ERBB3_226213_at	1.25	2.6×10^{-1}
JAK3_211109_at	1.24	2.8×10^{-1}
ERBB3_1563253_s_at	1.23	2.9×10^{-1}
SRC_1565082_x_at	1.2	3.6×10^{-1}
ERBB1_211551_at	1.18	4.0×10^{-1}
BTK_205504_at	1.08	6.9×10^{-1}
PTK2_241453_at	1.07	7.4×10^{-1}
ERBB2_216836_s_at	1	9.9×10^{-1}
ERBB3_1563252_at	0.96	8.2×10^{-1}
ERBB1_201983_s_at	0.89	5.7×10^{-1}
PTK2_207821_s_at	0.83	3.5×10^{-1}
PTK2_1559529_at	0.82	3.2×10^{-1}
MERTK_206028_s_at	0.75	1.5×10^{-1}
ERBB2_210930_s_at	0.74	1.3×10^{-1}
JAK2_205841_at	0.68	5.2×10^{-2}
SRC_1558210_at	0.67	4.5×10^{-2}
SRC_213324_at	0.64	2.5×10^{-2}
FLT3_206674_at	0.62	1.8×10^{-2}
SRC_221284_s_at	0.62	1.6×10^{-2}
BMX_206464_at	0.6	1.1×10^{-2}
SRC_1558211_s_at	0.59	8.0×10^{-3}
JAK1_201648_at	0.58	5.9×10^{-3}
PTK2_208820_at	0.57	5.2×10^{-3}
TEC_206301_at	0.53	1.3×10^{-3}
BLK_210934_at	0.52	1.0×10^{-3}
JAK3_211108_s_at	0.49	3.4×10^{-4}
LYN_202626_s_at	0.48	2.5×10^{-4}
BLK_206255_at	0.41	6.3×10^{-6}
TYK2_205546_s_at	0.4	4.3×10^{-6}
JAK3_207187_at	0.37	5.5×10^{-7}
LCK_204890_s_at	0.34	4.7×10^{-8}
JAK2_205842_s_at	0.32	$<1 \times 10^{-8}$
LCK_204891_s_at	0.32	$<1 \times 10^{-8}$
FYN_212486_s_at	0.3	$<1 \times 10^{-8}$
JAK1_239695_at	0.27	$<1 \times 10^{-8}$
SYK_207540_s_at	0.27	$<1 \times 10^{-8}$
JAK3_227677_at	0.26	$<1 \times 10^{-8}$
LYN_210754_s_at	0.25	$<1 \times 10^{-8}$
MERTK_211913_s_at	0.25	$<1 \times 10^{-8}$

LYN_202625_at	0.25	$<1 \times 10^{-8}$
JAK2_1562031_at	0.21	$<1 \times 10^{-8}$
SYK_209269_s_at	0.19	$<1 \times 10^{-8}$
FYN_210105_s_at	0.18	$<1 \times 10^{-8}$
HCK_208018_s_at	0.15	$<1 \times 10^{-8}$
SYK_226068_at	0.14	$<1 \times 10^{-8}$
JAK1_240613_at	0.12	$<1 \times 10^{-8}$
FYN_216033_s_at	0.11	$<1 \times 10^{-8}$
JAK1_1552610_a_at	0.08	$<1 \times 10^{-8}$
SYK_244023_at	0.07	$<1 \times 10^{-8}$
JAK1_1552611_a_at	0.06	$<1 \times 10^{-8}$
FGR_208438_s_at	0.03	$<1 \times 10^{-8}$

Table S2. Descriptive statistics for raw ERBB1 gene expression levels in malignant plasma cells from SKY-92 risk-assessed MM patients and normal CD138$^+$ plasma cells from healthy subjects

Probeset	Normal CD138$^+$ Plasma Cells (N=7)		SKY-92 Standard Risk (N=219)		SKY-92 High Risk (N=63)	
	Mean ± SEM	Median (Range)	Mean ± SEM	Median (Range)	Mean ± SEM	Median (Range)
EGFR/ERBB1_1565483_at	3.78 ± 0.42	3.19 (3.01 - 5.42)	7.74 ± 0.13	7.04 (5.1 - 11.7)	8.27 ± 0.23	8.37 (5.33 - 11.57)
EGFR/ERBB1_1565484_x_at	3.47 ± 0.38	2.9 (2.81 - 4.95)	6.7 ± 0.1	6.3 (4.47 - 10.15)	7.05 ± 0.17	7.07 (4.5 - 9.72)
EGFR/ERBB1_201983_s_at	4.55 ± 0.35	4.26 (3.74 - 6.6)	4.41 ± 0.02	4.35 (3.8 - 5.84)	4.32 ± 0.03	4.34 (3.86 - 5.13)
EGFR/ERBB1_201984_s_at	4.67 ± 0.23	4.89 (3.57 - 5.14)	5.46 ± 0.03	5.41 (4.55 - 6.95)	5.49 ± 0.04	5.53 (4.61 - 6.06)
EGFR/ERBB1_210984_x_at	4.95 ± 0.28	4.66 (4.16 - 6.03)	6.18 ± 0.03	6.12 (5.17 - 7.54)	6.16 ± 0.06	6.18 (4.93 - 7.04)
EGFR/ERBB1_211550_at	5.17 ± 0.07	5.23 (4.9 - 5.42)	5.54 ± 0.02	5.48 (4.72 - 6.69)	5.56 ± 0.03	5.57 (4.89 - 6.17)
EGFR/ERBB1_211551_at	5.18 ± 0.09	5.13 (4.92 - 5.59)	5.42 ± 0.02	5.4 (4.66 - 6.4)	5.39 ± 0.04	5.37 (4.75 - 6.13)
EGFR/ERBB1_211607_x_at	4.75 ± 0.27	4.65 (3.97 - 5.75)	6.1 ± 0.03	6.09 (4.94 - 7.49)	6.1 ± 0.06	6.02 (4.67 - 7.14)
EGFR/ERBB1_224999_at	4.84 ± 0.12	4.88 (4.37 - 5.14)	5.29 ± 0.03	5.23 (4.56 - 7.18)	5.25 ± 0.04	5.22 (4.67 - 7.11)
ERBB2_210930_s_at	5.17 ± 0.33	4.84 (4.27 - 6.78)	4.76 ± 0.03	4.69 (3.73 - 6.33)	4.63 ± 0.05	4.58 (3.75 - 6.26)
ERBB2_216836_s_at	5.96 ± 0.19	6.06 (5.18 - 6.46)	5.97 ± 0.04	5.86 (5.24 - 11.58)	5.97 ± 0.04	5.95 (5.28 - 6.94)
ERBB2_234354_x_at	3.9 ± 0.08	3.81 (3.65 - 4.22)	4.33 ± 0.03	4.28 (3.75 - 10.24)	4.25 ± 0.03	4.23 (3.83 - 4.81)
ERBB3_1563252_at	5.07 ± 0.1	5.03 (4.65 - 5.57)	5.02 ± 0.02	4.96 (4.36 - 6.38)	4.94 ± 0.04	4.9 (4.41 - 6.2)
ERBB3_1563253_s_at	5.06 ± 0.19	5.23 (4.47 - 5.67)	5.36 ± 0.02	5.33 (4.61 - 6.25)	5.4 ± 0.04	5.44 (4.82 - 6.01)
ERBB3_202454_s_at	3.66 ± 0.16	3.57 (3.29 - 4.33)	4.29 ± 0.02	4.26 (3.63 - 5.17)	4.29 ± 0.04	4.3 (3.71 - 5)
ERBB3_215638_at	4.05 ± 0.06	4.07 (3.85 - 4.33)	5.08 ± 0.03	5 (4.24 - 6.69)	5.02 ± 0.04	4.95 (4.22 - 6.05)
ERBB3_226213_at	4.01 ± 0.05	4.02 (3.75 - 4.17)	4.33 ± 0.02	4.28 (3.79 - 5.94)	4.34 ± 0.03	4.32 (3.86 - 5.3)

Table S3. Descriptive statistics for ERBB1 probesets in malignant plasma cells from newly diagnosed MM patients risk assessed using ISS staging criteria

Probeset	Stage I MM patients (N=109)		Stage II MM patients (N=70)		Stage III MM patients (N=77)	
	Mean ± SEM	Median (Range)	Mean ± SEM	Median (Range)	Mean ± SEM	Median (Range)
EGFR/ERBB1_1565483_at	7.69 ± 0.18	7.03 (5.12 - 11.58)	7.9 ± 0.21	7.88 (5.11 - 11.6)	8.04 ± 0.21	7.59 (5.43 - 11.7)
EGFR/ERBB1_1565484_x_at	6.63 ± 0.14	6.07 (4.47 - 9.98)	6.76 ± 0.15	6.63 (4.71 - 9.65)	6.99 ± 0.15	6.73 (4.92 - 10.15)
EGFR/ERBB1_201983_s_at	4.42 ± 0.03	4.4 (3.93 - 5.84)	4.32 ± 0.03	4.31 (3.81 - 4.82)	4.38 ± 0.03	4.34 (3.93 - 5.41)
EGFR/ERBB1_201984_s_at	5.44 ± 0.03	5.39 (4.86 - 6.65)	5.45 ± 0.04	5.48 (4.75 - 6.3)	5.51 ± 0.04	5.52 (4.55 - 6.48)
EGFR/ERBB1_210984_x_at	6.19 ± 0.04	6.11 (5.17 - 7.54)	6.16 ± 0.05	6.15 (5.23 - 7.04)	6.17 ± 0.06	6.16 (4.93 - 7.25)
EGFR/ERBB1_211550_at	5.54 ± 0.03	5.47 (4.95 - 6.69)	5.51 ± 0.03	5.52 (4.72 - 6.16)	5.58 ± 0.03	5.54 (5.04 - 6.47)
EGFR/ERBB1_211551_at	5.44 ± 0.03	5.42 (4.66 - 6.25)	5.38 ± 0.04	5.35 (4.68 - 6.08)	5.43 ± 0.04	5.41 (4.84 - 6.4)
EGFR/ERBB1_211607_x_at	6.12 ± 0.05	6.13 (5.3 - 7.49)	6.07 ± 0.06	6.08 (4.94 - 7.21)	6.12 ± 0.06	6.04 (5.22 - 7.29)
EGFR/ERBB1_224999_at	5.26 ± 0.04	5.22 (4.67 - 6.79)	5.25 ± 0.03	5.24 (4.65 - 6.28)	5.31 ± 0.04	5.23 (4.56 - 7.11)
ERBB2_210930_s_at	4.8 ± 0.04	4.77 (3.94 - 6.32)	4.61 ± 0.05	4.54 (3.96 - 6.21)	4.69 ± 0.06	4.6 (3.73 - 6.28)
ERBB2_216836_s_at	5.94 ± 0.04	5.87 (5.31 - 7.3)	5.89 ± 0.04	5.86 (5.28 - 6.9)	5.95 ± 0.04	5.9 (5.24 - 7.26)
ERBB2_234354_x_at	4.32 ± 0.02	4.3 (3.89 - 4.82)	4.27 ± 0.03	4.24 (3.75 - 4.8)	4.29 ± 0.03	4.3 (3.8 - 4.81)
ERBB3_1563252_at	5.01 ± 0.03	4.92 (4.54 - 6.06)	4.94 ± 0.03	4.97 (4.39 - 5.76)	5 ± 0.04	4.96 (4.41 - 6.22)
ERBB3_1563253_s_at	5.3 ± 0.03	5.29 (4.61 - 6.21)	5.38 ± 0.03	5.39 (4.93 - 6.11)	5.43 ± 0.04	5.43 (4.7 - 6.18)
ERBB3_202454_s_at	4.33 ± 0.03	4.29 (3.67 - 5.17)	4.24 ± 0.03	4.24 (3.74 - 4.87)	4.29 ± 0.04	4.3 (3.73 - 5.1)
ERBB3_215638_at	5.06 ± 0.04	4.94 (4.24 - 6.6)	5.04 ± 0.04	5.02 (4.51 - 6.47)	5.08 ± 0.05	5.04 (4.48 - 6.69)
ERBB3_226213_at	4.33 ± 0.03	4.28 (3.79 - 5.53)	4.28 ± 0.02	4.29 (3.87 - 4.66)	4.37 ± 0.03	4.33 (3.81 - 5.52)

Table S4. Gene expression levels for tyrosine kinases in malignant plasma cells from high-risk MM patients vs. normal plasma cells from healthy subjects.

Probeset	Fold Change (High-Risk/Control)	Linear Contrast (P-value)
ERBB1_1565483_at	22.39	$<1 \times 10^{-8}$
ERBB1_1565484_x_at	11.94	$<1 \times 10^{-8}$
ERBB1_211607_x_at	2.55	1.9×10^{-6}

BCMA_206641_at	2.34	1.5 x 10^{-5}
ERBB1_210984_x_at	2.32	1.9 x 10^{-5}
ERBB3_215638_at	1.95	6.5 x 10^{-4}
SRC_1565080_at	1.85	1.7 x 10^{-3}
ERBB1_201984_s_at	1.76	4.1 x 10^{-3}
MERTK_211912_at	1.66	9.8 x 10^{-3}
ERBB3_202454_s_at	1.54	2.7 x 10^{-2}
SRC_221281_at	1.36	1.2 x 10^{-1}
ERBB1_224999_at	1.33	1.4 x 10^{-1}
ERBB1_211550_at	1.31	1.7 x 10^{-1}
ERBB2_234354_x_at	1.28	2.1 x 10^{-1}
ERBB3_1563253_s_at	1.26	2.4 x 10^{-1}
ERBB3_226213_at	1.26	2.4 x 10^{-1}
SRC_1565082_x_at	1.21	3.2 x 10^{-1}
ERBB1_211551_at	1.16	4.5 x 10^{-1}
JAK3_211109_at	1.15	4.7 x 10^{-1}
BTK_205504_at	1.07	7.3 x 10^{-1}
PTK2_241453_at	1.05	8.0 x 10^{-1}
ERBB2_216836_s_at	1	9.9 x 10^{-1}
ERBB3_1563252_at	0.91	6.5 x 10^{-1}
ERBB1_201983_s_at	0.85	4.1 x 10^{-1}
PTK2_1559529_at	0.81	2.9 x 10^{-1}
PTK2_207821_s_at	0.79	2.2 x 10^{-1}
JAK2_205841_at	0.72	9.0 x 10^{-2}
ERBB2_210930_s_at	0.69	5.6 x 10^{-2}
SRC_1558210_at	0.64	2.4 x 10^{-2}
SRC_213324_at	0.61	1.2 x 10^{-2}
BMX_206464_at	0.61	1.2 x 10^{-2}
JAK1_201648_at	0.6	9.3 x 10^{-3}
SRC_221284_s_at	0.59	7.4 x 10^{-3}
SRC_1558211_s_at	0.59	6.3 x 10^{-3}
TEC_206301_at	0.54	1.6 x 10^{-3}
FLT3_206674_at	0.52	9.5 x 10^{-4}
MERTK_206028_s_at	0.51	6.1 x 10^{-4}
BLK_210934_at	0.51	5.7 x 10^{-4}
JAK3_211108_s_at	0.5	3.8 x 10^{-4}
LYN_202626_s_at	0.49	2.8 x 10^{-4}
PTK2_208820_at	0.47	1.3 x 10^{-4}
TYK2_205546_s_at	0.45	4.3 x 10^{-5}
BLK_206255_at	0.44	3.6 x 10^{-5}
JAK3_207187_at	0.36	2.6 x 10^{-7}
JAK3_227677_at	0.36	1.5 x 10^{-7}
JAK2_205842_s_at	0.34	2.8 x 10^{-8}
LCK_204890_s_at	0.31	<1 x 10^{-8}
LYN_210754_s_at	0.28	<1 x 10^{-8}
FYN_212486_s_at	0.28	<1 x 10^{-8}
JAK1_239695_at	0.27	<1 x 10^{-8}
SYK_207540_s_at	0.27	<1 x 10^{-8}
LCK_204891_s_at	0.26	<1 x 10^{-8}
LYN_202625_at	0.26	<1 x 10^{-8}
MERTK_211913_s_at	0.22	<1 x 10^{-8}
FYN_210105_s_at	0.21	<1 x 10^{-8}
JAK2_1562031_at	0.2	<1 x 10^{-8}
SYK_209269_s_at	0.19	<1 x 10^{-8}
SYK_226068_at	0.14	<1 x 10^{-8}
HCK_208018_s_at	0.13	<1 x 10^{-8}
FYN_216033_s_at	0.12	<1 x 10^{-8}
JAK1_240613_at	0.12	<1 x 10^{-8}

JAK1_1552610_a_at	0.09	$<1 \times 10^{-8}$
SYK_244023_at	0.07	$<1 \times 10^{-8}$
JAK1_1552611_a_at	0.05	$<1 \times 10^{-8}$
FGR_208438_s_at	0.03	$<1 \times 10^{-8}$

Table S5. Gene expression levels for tyrosine kinases in malignant plasma cells from high-risk vs. standard-risk MM patients

Probeset	Fold Change (High-Risk/Standard Risk)	Linear Contrast (P-value)
JAK3_227677_at	1.48	1.4×10^{-7}
ERBB1_1565483_at	1.44	9.0×10^{-7}
ERBB1_1565484_x_at	1.27	1.2×10^{-3}
FYN_210105_s_at	1.21	1.2×10^{-2}
JAK1_1552610_a_at	1.17	3.2×10^{-2}
LYN_210754_s_at	1.16	5.2×10^{-2}
TYK2_205546_s_at	1.15	5.5×10^{-2}
BLK_206255_at	1.12	1.3×10^{-1}
FYN_216033_s_at	1.1	2.0×10^{-1}
JAK2_205841_at	1.07	3.4×10^{-1}
JAK2_205842_s_at	1.07	3.7×10^{-1}
LYN_202625_at	1.06	4.6×10^{-1}
JAK1_201648_at	1.05	5.1×10^{-1}
ERBB3_1563253_s_at	1.03	7.3×10^{-1}
TEC_206301_at	1.02	7.4×10^{-1}
ERBB1_201984_s_at	1.02	7.9×10^{-1}
LYN_202626_s_at	1.02	7.9×10^{-1}
JAK3_211108_s_at	1.02	8.0×10^{-1}
BMX_206464_at	1.02	8.2×10^{-1}
SRC_1565082_x_at	1.02	8.3×10^{-1}
JAK1_239695_at	1.02	8.4×10^{-1}
ERBB1_211550_at	1.01	8.4×10^{-1}
FGR_208438_s_at	1.01	9.3×10^{-1}
ERBB3_226213_at	1.01	9.3×10^{-1}
SYK_207540_s_at	1.01	9.4×10^{-1}
ERBB1_211607_x_at	1	9.6×10^{-1}
ERBB2_216836_s_at	1	9.9×10^{-1}
ERBB3_202454_s_at	1	1.0×10^{0}
SYK_209269_s_at	0.99	9.4×10^{-1}
ERBB1_210984_x_at	0.99	9.0×10^{-1}
PTK2_1559529_at	0.99	8.9×10^{-1}
SYK_244023_at	0.99	8.9×10^{-1}
SRC_1558211_s_at	0.99	8.9×10^{-1}
SRC_1565080_at	0.99	8.8×10^{-1}
JAK1_240613_at	0.99	8.6×10^{-1}
BTK_205504_at	0.98	8.4×10^{-1}
PTK2_241453_at	0.98	7.8×10^{-1}
JAK3_207187_at	0.98	7.8×10^{-1}
ERBB1_211551_at	0.98	7.6×10^{-1}
ERBB1_224999_at	0.97	7.2×10^{-1}
BLK_210934_at	0.97	6.9×10^{-1}
BCMA_206641_at	0.97	6.5×10^{-1}
ERBB3_215638_at	0.96	5.9×10^{-1}
SYK_226068_at	0.96	5.8×10^{-1}
SRC_221281_at	0.96	5.8×10^{-1}
JAK2_1562031_at	0.96	5.4×10^{-1}
MERTK_211912_at	0.95	5.3×10^{-1}
ERBB2_234354_x_at	0.95	4.8×10^{-1}
ERBB3_1563252_at	0.95	4.5×10^{-1}
SRC_1558210_at	0.94	4.4×10^{-1}

SRC_221284_s_at	0.94	4.1	x 10	$^{-1}$
SRC_213324_at	0.94	4.0	x 10	$^{-1}$
ERBB1_201983_s_at	0.94	4.0	x 10	$^{-1}$
JAK1_1552611_a_at	0.93	3.3	x 10	$^{-1}$
PTK2_207821_s_at	0.93	3.2	x 10	$^{-1}$
FYN_212486_s_at	0.91	2.3	x 10	$^{-1}$
ERBB2_210930_s_at	0.91	2.0	x 10	$^{-1}$
JAK3_211109_at	0.91	2.0	x 10	$^{-1}$
LCK_204890_s_at	0.9	1.5	x 10	$^{-1}$
MERTK_211913_s_at	0.86	4.5	x 10	$^{-2}$
HCK_208018_s_at	0.84	2.0	x 10	$^{-2}$
FLT3_206674_at	0.8	2.1	x 10	$^{-3}$
LCK_204891_s_at	0.79	1.2	x 10	$^{-3}$
PTK2_208820_at	0.78	7.7	x 10	$^{-4}$
MERTK_206028_s_at	0.61	<1	x 10	$^{-8}$

Table S6. Correlated expression of transcription factor genes with EGFR/ERBB1 gene expression (Probeset: EGFR_1565484_x_at) in MM patients

	ERBB1^{high} MM patients (N=94)		ERBB1^{low} MM patients (N=94)		Pearson Correlation (N=282)		ERBB1^{high} versus ERBB1^{low}	
Probeset	Mean ± SEM	Median (Range)	Mean ± SEM	Median (Range)	Coefficient	P-value	Fold Change	P-value
CTNNB1_1554411_at	4.29 ± 0.03	4.24 (3.82 - 5.56)	4.02 ± 0.03	3.97 (3.42 - 5.26)	0.42	$9.1x10^{-14}$	1.21	$<1x10^{-6}$
CTNNB1_201533_at	7.43 ± 0.07	7.43 (4.93 - 9.43)	8.07 ± 0.09	8.03 (4.98 - 9.97)	−0.33	$1.1x10^{-8}$	0.64	$<1x10^{-6}$
CTNNB1_223679_at	5.72 ± 0.07	5.75 (4.01 - 7.75)	6.08 ± 0.08	6.01 (4.27 - 8.51)	−0.21	$2.9x10^{-4}$	0.78	$6.3x10^{-4}$
EGFR/ERBB1_1565483_at	10.12 ± 0.1	10.29 (8.28 - 11.7)	6 ± 0.04	6 (5.1 - 6.77)	0.98	$6.0x10^{-210}$	17.33	$<1x10^{-6}$
EGFR/ERBB1_1565484_x_at	8.47 ± 0.08	8.52 (7.3 - 10.15)	5.38 ± 0.04	5.42 (4.47 - 5.86)	1	NA	8.52	$<1x10^{-6}$
EGFR/ERBB1_201983_s_at	4.56 ± 0.04	4.47 (4.1 - 5.84)	4.31 ± 0.02	4.31 (3.8 - 4.82)	0.45	$1.4x10^{-15}$	1.19	$<1x10^{-6}$
EGFR/ERBB1_201984_s_at	5.73 ± 0.04	5.66 (4.97 - 6.95)	5.22 ± 0.03	5.22 (4.55 - 5.88)	0.62	$8.5x10^{-32}$	1.42	$<1x10^{-6}$
EGFR/ERBB1_210984_x_at	6.5 ± 0.05	6.5 (4.93 - 7.54)	5.93 ± 0.04	5.97 (5.01 - 6.69)	0.56	$3.8x10^{-25}$	1.48	$<1x10^{-6}$
EGFR/ERBB1_211550_at	5.77 ± 0.03	5.7 (5.32 - 6.69)	5.37 ± 0.02	5.35 (4.72 - 6.06)	0.65	$8.7x10^{-35}$	1.32	$<1x10^{-6}$
EGFR/ERBB1_211551_at	5.56 ± 0.03	5.5 (4.88 - 6.4)	5.36 ± 0.03	5.36 (4.68 - 6.14)	0.36	$4.9x10^{-10}$	1.15	$2.4x10^{-5}$
EGFR/ERBB1_211607_x_at	6.47 ± 0.05	6.45 (5.21 - 7.49)	5.81 ± 0.04	5.77 (4.67 - 6.95)	0.63	$3.3x10^{-32}$	1.59	$<1x10^{-6}$
EGFR/ERBB1_224999_at	5.57 ± 0.05	5.39 (4.87 - 7.18)	5.08 ± 0.02	5.09 (4.56 - 5.55)	0.65	$2.5x10^{-35}$	1.4	$<1x10^{-6}$
FOS_209189_at	10.27 ± 0.11	10.48 (7.31 - 12.33)	11.18 ± 0.16	11.42 (6.62 - 13.56)	−0.29	$1.0x10^{-6}$	0.53	$5.0x10^{-6}$
FOSB_202768_at	10.23 ± 0.12	10.33 (7.36 - 12.56)	10.33 ± 0.18	10.31 (6.57 - 13.4)	−0.07	$2.4x10^{-1}$	0.94	$6.5x10^{-1}$
FOSL1_204420_at	5.96 ± 0.06	5.82 (5 - 7.9)	5.35 ± 0.03	5.35 (4.57 - 6.16)	0.63	$4.2x10^{-32}$	1.52	$<1x10^{-6}$
FOSL2_205409_at	5.85 ± 0.05	5.76 (4.91 - 7.11)	5.58 ± 0.04	5.57 (4.6 - 6.28)	0.39	$1.5x10^{-11}$	1.21	$5.9x10^{-5}$
FOSL2_218880_at	4.58 ± 0.06	4.49 (3.82 - 7.09)	4.53 ± 0.06	4.41 (3.82 - 7.47)	0.08	$2.1x10^{-1}$	1.04	$5.2x10^{-1}$
FOSL2_218881_s_at	5.74 ± 0.04	5.74 (4.75 - 7.17)	5.44 ± 0.03	5.45 (4.72 - 6.41)	0.45	$4.0x10^{-15}$	1.23	$<1x10^{-6}$
FOSL2_225262_at	6.05 ± 0.09	6.08 (4.47 - 9.19)	6.14 ± 0.09	5.87 (4.74 - 9.08)	−0.1	$1.0x10^{-1}$	0.94	$5.0x10^{-1}$
FOSL2_228188_at	4.58 ± 0.06	4.48 (3.8 - 6.49)	4.51 ± 0.05	4.49 (3.74 - 6.87)	0.09	$1.3x10^{-1}$	1.05	$3.5x10^{-1}$
GCFC2_210175_at	6.8 ± 0.07	6.66 (5.46 - 10.08)	6.96 ± 0.08	6.87 (5.43 - 9.17)	−0.12	$4.1x10^{-2}$	0.89	$1.3x10^{-1}$
GCFC2_216305_s_at	5.23 ± 0.06	5.16 (4.31 - 6.86)	5.12 ± 0.07	4.98 (3.95 - 6.92)	0.12	$5.2x10^{-2}$	1.08	$2.3x10^{-1}$
HOXB5_205600_x_at	5.59 ± 0.03	5.56 (5.02 - 6.77)	5.22 ± 0.03	5.21 (4.65 - 5.74)	0.55	$3.6x10^{-24}$	1.29	$<1x10^{-6}$
HOXB5_205601_s_at	6.11 ± 0.03	6.13 (5.42 - 6.9)	5.95 ± 0.03	5.97 (5.16 - 6.58)	0.33	$1.1x10^{-8}$	1.11	$1.9x10^{-4}$
IRF1_202531_at	8.12 ± 0.06	8.18 (6.57 - 9.84)	8.71 ± 0.1	8.7 (6.09 - 10.99)	−0.29	$1.1x10^{-6}$	0.66	$1.0x10^{-6}$
IRF1_238725_at	8.53 ± 0.07	8.49 (6.46 - 10.19)	8.68 ± 0.12	8.49 (5.59 - 11.83)	−0.09	$1.1x10^{-1}$	0.9	$2.8x10^{-1}$
JUN_201464_x_at	10.17 ± 0.07	10.23 (8.66 - 11.73)	10.86 ± 0.09	11.03 (8.36 - 12.23)	−0.38	$6.2x10^{-11}$	0.62	$<1x10^{-6}$
JUN_201465_s_at	8.74 ± 0.1	8.74 (6.25 - 10.88)	9.44 ± 0.15	9.4 (5.76 - 12.65)	−0.25	$2.6x10^{-5}$	0.62	$2.1x10^{-4}$
JUN_201466_s_at	9.65 ± 0.09	9.68 (7.53 - 11.63)	10.26 ± 0.15	10.48 (6.78 - 12.78)	−0.24	$4.8x10^{-5}$	0.65	$7.1x10^{-4}$
JUN_213281_at	6.9 ± 0.07	7 (5.31 - 8.16)	7 ± 0.1	7.12 (4.93 - 9.62)	−0.07	$2.2x10^{-1}$	0.94	$4.4x10^{-1}$
JUNB_201473_at	8.68 ± 0.07	8.72 (7.37 - 10.21)	9.34 ± 0.09	9.26 (6.74 - 11.28)	−0.35	$2.5x10^{-9}$	0.63	$<1x10^{-6}$
JUND_203751_x_at	6.13 ± 0.08	6.01 (4.63 - 10.18)	6.15 ± 0.11	5.85 (4.56 - 9.66)	0.05	$4.3x10^{-1}$	0.98	$8.6x10^{-1}$
JUND_203752_s_at	10.86 ± 0.08	10.86 (9.55 - 13.24)	12.11 ± 0.08	12.21 (9.78 - 13.52)	−0.59	$2.7x10^{-28}$	0.42	$<1x10^{-6}$
JUND_214326_x_at	5.55 ± 0.09	5.33 (4.22 - 9.43)	5.55 ± 0.12	5.22 (3.95 - 9.36)	0.07	$2.4x10^{-1}$	1	$9.9x10^{-1}$
JUND_229117_s_at	5.16 ± 0.05	5.04 (4.29 - 6.68)	5.04 ± 0.04	4.98 (4.28 - 6.75)	0.27	$4.5x10^{-6}$	1.09	$5.1x10^{-2}$
LEF1_210948_s_at	4.8 ± 0.05	4.66 (3.93 - 6.33)	4.5 ± 0.07	4.41 (3.63 - 8.83)	0.31	$1.4x10^{-7}$	1.23	$4.4x10^{-4}$
LEF1_221557_s_at	5.83 ± 0.04	5.78 (5.1 - 6.74)	5.55 ± 0.03	5.54 (4.97 - 6.38)	0.43	$2.2x10^{-14}$	1.21	$<1x10^{-6}$
LEF1_221558_s_at	6.19 ± 0.13	5.82 (4.34 - 9.89)	5.89 ± 0.16	5.34 (4.25 - 11.3)	0.1	$7.9x10^{-2}$	1.24	$1.4x10^{-1}$
POU6F2_207450_s_at	5.1 ± 0.05	4.99 (4.44 - 6.68)	4.69 ± 0.03	4.68 (4.1 - 5.37)	0.56	$6.9x10^{-25}$	1.33	$<1x10^{-6}$
SP1_1553685_s_at	3.92 ± 0.04	3.87 (3.34 - 5.37)	3.92 ± 0.04	3.88 (3.39 - 6.33)	0.09	$1.4x10^{-1}$	1	$9.1x10^{-1}$
SP1_214732_at	4.66 ± 0.04	4.6 (4.13 - 5.85)	4.38 ± 0.02	4.36 (3.92 - 4.86)	0.5	$1.6x10^{-19}$	1.21	$<1x10^{-6}$
SP1_224754_at	8.19 ± 0.05	8.15 (7.11 - 9.4)	8.49 ± 0.08	8.55 (6.49 - 10.9)	−0.19	$1.7x10^{-3}$	0.81	$1.3x10^{-3}$

SP1_224760_at	6.88 ± 0.06	6.91 (5.47 - 8.13)	6.54 ± 0.09	6.45 (4.45 - 8.6)	0.22	2.3x10^{-4}	1.26	1.5x10^{-3}
TBP_203135_at	6.64 ± 0.06	6.61 (5.45 - 8.07)	6.66 ± 0.09	6.7 (5.05 - 8.64)	−0.03	5.7x10^{-1}	0.99	8.5x10^{-1}
TCF7_205254_x_at	5.98 ± 0.04	5.88 (5.21 - 7.04)	5.59 ± 0.03	5.64 (4.83 - 6.05)	0.6	4.1x10^{-29}	1.31	<1x10^{-6}
TCF7_205255_x_at	7.21 ± 0.05	7.19 (6.2 - 8.32)	7.58 ± 0.05	7.51 (6.55 - 9.37)	−0.4	1.6x10^{-12}	0.77	<1x10^{-6}
TCF7L1_221016_s_at	5.66 ± 0.04	5.65 (4.8 - 7.21)	5.3 ± 0.03	5.29 (4.6 - 6.42)	0.5	3.3x10^{-19}	1.29	<1x10^{-6}
TCF7L2_212759_s_at	6.39 ± 0.07	6.15 (5.22 - 8.06)	5.67 ± 0.03	5.66 (4.8 - 6.82)	0.65	1.0x10^{-35}	1.65	<1x10^{-6}
TCF7L2_212761_at	5.99 ± 0.14	5.7 (3.96 - 9.61)	6.11 ± 0.17	5.65 (3.42 - 10.67)	−0.05	3.8x10^{-1}	0.92	5.8x10^{-1}
TCF7L2_212762_s_at	4.57 ± 0.09	4.23 (3.56 - 7.34)	4.63 ± 0.09	4.37 (3.48 - 7.07)	−0.02	7.9x10^{-1}	0.96	6.2x10^{-1}
TCF7L2_216035_x_at	5.55 ± 0.07	5.31 (4.72 - 8.39)	5.36 ± 0.09	5.06 (4.42 - 8.91)	0.14	2.3x10^{-2}	1.14	1.0x10^{-1}
TCF7L2_216037_x_at	5.77 ± 0.06	5.59 (5.03 - 7.08)	5.52 ± 0.06	5.39 (4.53 - 7.9)	0.22	2.0x10^{-4}	1.19	4.7x10^{-3}
TCF7L2_216511_s_at	4.58 ± 0.05	4.42 (3.92 - 6.42)	4.47 ± 0.06	4.32 (3.78 - 7.34)	0.11	6.6x10^{-2}	1.08	1.6x10^{-1}
TCF7L2_236094_at	5.36 ± 0.04	5.31 (4.75 - 6.61)	5.01 ± 0.03	5 (4.43 - 5.55)	0.56	7.9x10^{-25}	1.27	<1x10^{-6}
TEAD2_226408_at	5.6 ± 0.03	5.59 (4.91 - 6.78)	5.48 ± 0.03	5.44 (4.91 - 6.91)	0.23	1.2x10^{-4}	1.09	1.2x10^{-2}
TEAD2_238322_s_at	6.8 ± 0.05	6.72 (5.98 - 7.83)	6.29 ± 0.03	6.31 (5.33 - 6.82)	0.65	1.4x10^{-35}	1.42	<1x10^{-6}
TEAD2_238323_at	4.62 ± 0.04	4.53 (4.04 - 5.73)	4.36 ± 0.02	4.34 (3.81 - 4.87)	0.48	5.2x10^{-18}	1.2	<1x10^{-6}
TEAD2_243766_s_at	6.57 ± 0.03	6.61 (5.51 - 7.17)	6.71 ± 0.04	6.72 (5.71 - 7.65)	−0.08	1.9x10^{-1}	0.9	5.9x10^{-3}
TP53_201746_at	6.09 ± 0.07	6.12 (4.02 - 7.28)	6.47 ± 0.1	6.52 (4.34 - 8.74)	−0.14	2.1x10^{-2}	0.77	2.9x10^{-3}
TP53_211300_s_at	5.06 ± 0.03	5 (4.46 - 6.22)	5.16 ± 0.05	5.11 (4.16 - 6.75)	−0.02	7.0x10^{-1}	0.94	1.2x10^{-1}

NA: Not Applicable.

Table S7. Correlated expression of transcription factor genes with EGFR/ERBB1 gene expression (Probeset: EGFR_1565483_at) in MM patients

	ERBB1^{high} MM patients (N=94)		ERBB1^{low} MM patients (N=94)		Pearson Correlation (N=282)		ERBB1^{high} versus ERBB1^{low}	
Probeset	Mean ± SEM	Median (Range)	Mean ± SEM	Median (Range)	Coefficient	P-value	Fold Change	P-value
CTNNB1_1554411_at	4.28 ± 0.03	4.24 (3.82 - 5.56)	4.02 ± 0.03	3.97 (3.42 - 4.92)	0.4	4.5x10^{-12}	1.2	<1x10^{-6}
CTNNB1_201533_at	7.43 ± 0.07	7.39 (5.6 - 9.43)	8.03 ± 0.09	7.98 (4.98 - 9.97)	−0.33	1.3x10^{-8}	0.66	1.0x10^{-6}
CTNNB1_223679_at	5.68 ± 0.07	5.6 (4.01 - 7.75)	6.06 ± 0.08	6 (4.27 - 8.51)	−0.22	2.3x10^{-4}	0.77	3.0x10^{-4}
EGFR/ERBB1_1565483_at	10.15 ± 0.09	10.29 (8.77 - 11.7)	5.97 ± 0.04	6 (5.1 - 6.55)	1	NA	18.21	<1x10^{-6}
EGFR/ERBB1_1565484_x_at	8.43 ± 0.09	8.52 (6.57 - 10.15)	5.41 ± 0.04	5.42 (4.47 - 6.49)	0.98	6.0x10^{-210}	8.11	<1x10^{-6}
EGFR/ERBB1_201983_s_at	4.53 ± 0.04	4.46 (3.97 - 5.84)	4.3 ± 0.02	4.3 (3.8 - 4.82)	0.42	1.1x10^{-13}	1.18	1.0x10^{-6}
EGFR/ERBB1_201984_s_at	5.7 ± 0.04	5.63 (4.94 - 6.95)	5.21 ± 0.03	5.19 (4.55 - 5.85)	0.6	1.3x10^{-28}	1.4	<1x10^{-6}
EGFR/ERBB1_210984_x_at	6.46 ± 0.05	6.45 (4.93 - 7.54)	5.9 ± 0.03	5.92 (5.01 - 6.69)	0.55	1.9x10^{-23}	1.48	<1x10^{-6}
EGFR/ERBB1_211550_at	5.76 ± 0.03	5.7 (5.24 - 6.69)	5.35 ± 0.02	5.35 (4.72 - 5.86)	0.63	8.2x10^{-33}	1.33	<1x10^{-6}
EGFR/ERBB1_211551_at	5.55 ± 0.03	5.5 (4.92 - 6.4)	5.33 ± 0.03	5.35 (4.68 - 6.14)	0.35	1.9x10^{-9}	1.16	2.0x10^{-6}
EGFR/ERBB1_211607_x_at	6.45 ± 0.05	6.43 (5.21 - 7.49)	5.78 ± 0.04	5.73 (4.67 - 6.66)	0.62	5.5x10^{-31}	1.6	<1x10^{-6}
EGFR/ERBB1_224999_at	5.57 ± 0.05	5.4 (4.95 - 7.18)	5.08 ± 0.02	5.09 (4.56 - 5.55)	0.63	1.5x10^{-32}	1.4	<1x10^{-6}
FOS_209189_at	10.28 ± 0.12	10.46 (7.31 - 12.4)	11.13 ± 0.15	11.4 (6.62 - 13.56)	−0.27	4.7x10^{-6}	0.55	1.5x10^{-5}
FOSB_202768_at	10.34 ± 0.11	10.42 (7.63 - 12.56)	10.32 ± 0.18	10.35 (6.4 - 13.4)	−0.04	4.6x10^{-1}	1.02	9.1x10^{-1}
FOSL1_204420_at	5.94 ± 0.06	5.8 (4.8 - 7.9)	5.34 ± 0.03	5.32 (4.57 - 6.18)	0.61	5.4x10^{-30}	1.52	<1x10^{-6}
FOSL2_205409_at	5.85 ± 0.05	5.73 (4.91 - 7.11)	5.55 ± 0.04	5.56 (4.6 - 6.27)	0.38	3.9x10^{-11}	1.23	1.0x10^{-5}
FOSL2_218880_at	4.58 ± 0.06	4.5 (3.82 - 7.09)	4.54 ± 0.06	4.41 (3.82 - 7.47)	0.06	3.0x10^{-1}	1.03	6.4x10^{-1}
FOSL2_218881_s_at	5.74 ± 0.04	5.74 (4.75 - 7.17)	5.42 ± 0.03	5.43 (4.8 - 6.41)	0.43	2.3x10^{-14}	1.25	<1x10^{-6}
FOSL2_225262_at	6.09 ± 0.09	6.08 (4.47 - 9.19)	6.18 ± 0.09	5.91 (4.74 - 9.08)	−0.1	7.9x10^{-2}	0.94	4.7x10^{-1}
FOSL2_228188_at	4.59 ± 0.05	4.49 (3.8 - 6.49)	4.52 ± 0.05	4.49 (3.74 - 6.87)	0.08	1.8x10^{-1}	1.06	3.1x10^{-1}
GCFC2_210175_at	6.74 ± 0.07	6.62 (5.46 - 10.08)	7.03 ± 0.08	6.94 (5.43 - 9.17)	−0.15	1.4x10^{-2}	0.82	9.3x10^{-3}
GCFC2_216305_s_at	5.2 ± 0.06	5.13 (3.99 - 6.86)	5.15 ± 0.07	5.06 (3.95 - 6.92)	0.09	1.2x10^{-1}	1.03	6.2x10^{-1}

HOXB5_205600_x_at	5.57 ± 0.03	5.55 (5.02 - 6.77)	5.23 ± 0.03	5.23 (4.65 - 5.74)	0.54	2.4x10^{-22}	1.26	<1x10^{-6}
HOXB5_205601_s_at	6.11 ± 0.03	6.12 (5.42 - 6.9)	5.93 ± 0.03	5.96 (5.16 - 6.58)	0.32	3.5x10^{-8}	1.13	1.3x10^{-5}
IRF1_202531_at	8.12 ± 0.06	8.18 (6.57 - 9.84)	8.67 ± 0.1	8.72 (6.09 - 10.99)	−0.28	1.5x10^{-6}	0.68	2.0x10^{-6}
IRF1_238725_at	8.58 ± 0.07	8.56 (6.46 - 10.19)	8.65 ± 0.12	8.44 (5.59 - 11.83)	−0.09	1.4x10^{-1}	0.95	6.1x10^{-1}
JUN_201464_x_at	10.23 ± 0.08	10.23 (8.66 - 11.71)	10.84 ± 0.09	11 (8.36 - 12.25)	−0.36	3.8x10^{-10}	0.65	1.0x10^{-6}
JUN_201465_s_at	8.82 ± 0.1	8.87 (6.42 - 10.88)	9.49 ± 0.15	9.45 (5.76 - 12.65)	−0.23	9.6x10^{-5}	0.63	2.1x10^{-4}
JUN_201466_s_at	9.74 ± 0.09	9.8 (7.93 - 12.08)	10.27 ± 0.15	10.48 (6.78 - 12.78)	−0.22	1.5x10^{-4}	0.69	2.4x10^{-3}
JUN_213281_at	6.97 ± 0.07	7.02 (5.31 - 8.16)	7.01 ± 0.1	7.03 (4.93 - 9.62)	−0.06	2.8x10^{-1}	0.97	7.5x10^{-1}
JUNB_201473_at	8.67 ± 0.07	8.67 (7.28 - 10.02)	9.29 ± 0.09	9.21 (6.74 - 11.28)	−0.34	6.0x10^{-9}	0.65	<1x10^{-6}
JUND_203751_x_at	6.1 ± 0.08	5.97 (4.63 - 10.18)	6.11 ± 0.11	5.82 (4.35 - 9.66)	0.04	5.1x10^{-1}	0.99	9.4x10^{-1}
JUND_203752_s_at	10.88 ± 0.08	10.85 (9.55 - 13.24)	12.06 ± 0.08	12.18 (9.78 - 13.48)	−0.59	8.9x10^{-28}	0.44	<1x10^{-6}
JUND_214326_x_at	5.54 ± 0.09	5.29 (4.22 - 9.43)	5.51 ± 0.12	5.18 (4.04 - 9.36)	0.07	2.5x10^{-1}	1.02	8.8x10^{-1}
JUND_229117_s_at	5.19 ± 0.05	5.07 (4.32 - 6.68)	5.02 ± 0.04	4.98 (4.28 - 6.75)	0.26	1.3x10^{-5}	1.12	8.5x10^{-3}
LEF1_210948_s_at	4.78 ± 0.05	4.64 (3.93 - 6.33)	4.49 ± 0.07	4.4 (3.63 - 8.83)	0.3	2.0x10^{-7}	1.23	6.6x10^{-4}
LEF1_221557_s_at	5.84 ± 0.03	5.8 (5.1 - 6.74)	5.53 ± 0.03	5.52 (4.97 - 6.38)	0.43	2.2x10^{-14}	1.23	<1x10^{-6}
LEF1_221558_s_at	6.16 ± 0.13	5.79 (4.34 - 9.89)	5.93 ± 0.16	5.41 (4.25 - 11.3)	0.1	8.0x10^{-2}	1.17	2.7x10^{-1}
POU6F2_207450_s_at	5.09 ± 0.05	4.99 (4.44 - 6.68)	4.68 ± 0.03	4.67 (4.1 - 5.37)	0.54	1.2x10^{-22}	1.32	<1x10^{-6}
SP1_1553685_s_at	3.91 ± 0.04	3.84 (3.34 - 5.37)	3.9 ± 0.04	3.85 (3.29 - 6.33)	0.08	2.0x10^{-1}	1.01	8.5x10^{-1}
SP1_214732_at	4.64 ± 0.04	4.57 (4.1 - 5.85)	4.36 ± 0.02	4.35 (3.92 - 4.86)	0.47	7.1x10^{-17}	1.22	<1x10^{-6}
SP1_224754_at	8.21 ± 0.05	8.16 (7.24 - 9.4)	8.46 ± 0.08	8.51 (6.49 - 10.9)	−0.17	3.9x10^{-3}	0.84	7.2x10^{-3}
SP1_224760_at	6.88 ± 0.05	6.91 (5.62 - 8.03)	6.52 ± 0.08	6.45 (4.45 - 8.6)	0.22	1.7x10^{-4}	1.28	4.4x10^{-4}
TBP_203135_at	6.63 ± 0.05	6.59 (5.45 - 8.05)	6.65 ± 0.09	6.72 (5.05 - 8.64)	−0.03	6.6x10^{-1}	0.98	8.2x10^{-1}
TCF7_205254_x_at	5.97 ± 0.04	5.88 (5.21 - 7.04)	5.57 ± 0.02	5.61 (4.83 - 6.05)	0.59	1.1x10^{-27}	1.32	<1x10^{-6}
TCF7_205255_x_at	7.21 ± 0.05	7.21 (6.2 - 8.32)	7.6 ± 0.05	7.51 (6.55 - 9.37)	−0.41	9.6x10^{-13}	0.76	<1x10^{-6}
TCF7L1_221016_s_at	5.65 ± 0.05	5.63 (4.8 - 7.21)	5.27 ± 0.03	5.28 (4.6 - 6.02)	0.48	6.1x10^{-18}	1.29	<1x10^{-6}
TCF7L2_212759_s_at	6.35 ± 0.07	6.09 (5.22 - 8.06)	5.67 ± 0.04	5.65 (4.8 - 6.82)	0.64	8.9x10^{-34}	1.6	<1x10^{-6}
TCF7L2_212761_at	6.02 ± 0.13	5.79 (3.96 - 9.03)	6.16 ± 0.17	5.75 (3.42 - 10.67)	−0.04	5.5x10^{-1}	0.91	5.1x10^{-1}
TCF7L2_212762_s_at	4.55 ± 0.09	4.24 (3.56 - 7.29)	4.65 ± 0.09	4.33 (3.48 - 7.97)	−0.01	8.7x10^{-1}	0.93	4.4x10^{-1}
TCF7L2_216035_x_at	5.53 ± 0.07	5.32 (4.75 - 7.35)	5.38 ± 0.09	5.06 (4.42 - 8.91)	0.14	1.6x10^{-2}	1.11	1.6x10^{-1}
TCF7L2_216037_x_at	5.75 ± 0.06	5.57 (5.03 - 7.08)	5.51 ± 0.06	5.38 (4.53 - 7.9)	0.23	8.8x10^{-5}	1.18	5.3x10^{-3}
TCF7L2_216511_s_at	4.57 ± 0.05	4.47 (3.83 - 5.88)	4.49 ± 0.06	4.34 (3.78 - 7.34)	0.12	4.4x10^{-2}	1.06	2.6x10^{-1}
TCF7L2_236094_at	5.34 ± 0.04	5.31 (4.75 - 6.61)	4.98 ± 0.02	4.96 (4.43 - 5.47)	0.55	8.4x10^{-24}	1.28	<1x10^{-6}
TEAD2_226408_at	5.59 ± 0.03	5.56 (4.91 - 6.78)	5.47 ± 0.03	5.44 (4.91 - 6.91)	0.22	1.5x10^{-4}	1.08	1.4x10^{-2}
TEAD2_238322_s_at	6.8 ± 0.05	6.7 (5.99 - 7.83)	6.26 ± 0.03	6.29 (5.33 - 6.82)	0.64	1.6x10^{-34}	1.45	<1x10^{-6}
TEAD2_238323_at	4.62 ± 0.04	4.53 (4.07 - 5.73)	4.34 ± 0.02	4.31 (3.82 - 4.87)	0.46	2.1x10^{-16}	1.21	<1x10^{-6}
TEAD2_243766_s_at	6.57 ± 0.03	6.6 (5.51 - 7.17)	6.69 ± 0.04	6.71 (5.71 - 7.65)	−0.08	1.8x10^{-1}	0.92	1.8x10^{-2}
TP53_201746_at	6.04 ± 0.07	6.08 (4.02 - 7.28)	6.38 ± 0.11	6.39 (4.34 - 8.74)	−0.12	4.7x10^{-2}	0.79	9.8x10^{-3}
TP53_211300_s_at	5.03 ± 0.03	4.98 (4.46 - 6.22)	5.13 ± 0.05	5.08 (4.16 - 6.75)	−0.03	6.5x10^{-1}	0.94	1.2x10^{-1}

NA: Not Applicable.

Supplementary Figures

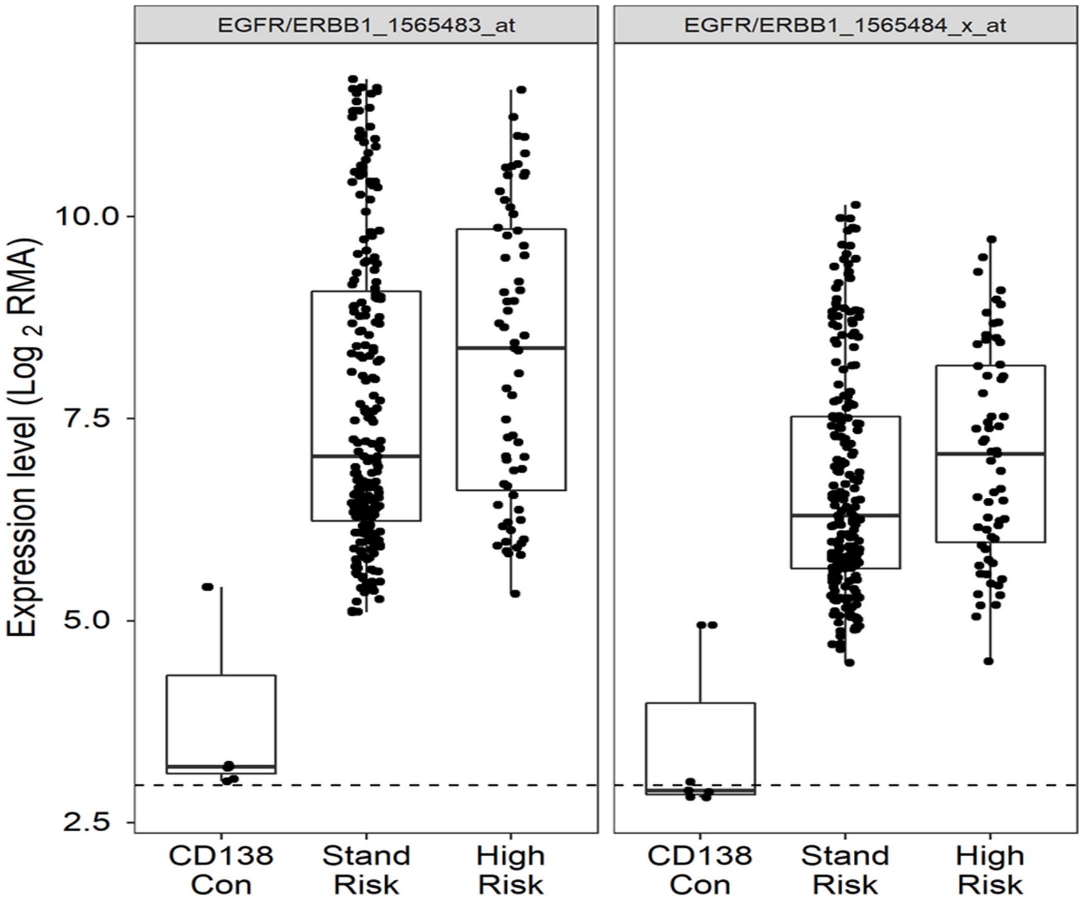

Figure S1. Absolute ERBB1 Gene Expression Levels in Malignant Plasma Cells from SKY-92 Risk-assessed MM Patients and Normal CD138[+] Plasma Cells from Healthy Subjects.We examined the gene expression data in the archived MM dataset GSE19784 and the control plasma cell dataset from GSE171739. We examined and compared the normalized log_2 RMA expression values for the ERBB1 gene expression levels for the two ERBB1 probesets: EGFR/ERBB1_1565483_at and EGFR/ERBB1_1565484_x_at, in CD138[+] control plasma cells (CD138 Con, N=7; GSE171739), malignant plasma cells from high-risk MM patients (N=63; GSE19784) and malignant plasma cells from standard-risk ("StandRisk") MM patients (N=219; GSE19784). The gene expression levels for the 2 probesets (filled circles) are depicted in the box-plot that represents the median expression (horizontal line in the box), the 75th and 25th quantile box, and the whiskers for the 3rd quartile + 1.5*(interquartile range) and 1st quartile - 1.5*(interquartile range) of the expression values. We compared the expression values of the standard risk and high-risk MM samples with the upper 95% confidence interval for the lowest expressing 100 probesets obtained from 7 control samples (dashed line = 3.0; Mean = 2.89 ± 0.04). The EGFR/ERBB1 gene expression levels for both probesets were above the lowest expressed transcripts in all MM samples. The mean ± SEM and median (Range) expression values for the EGFR/ERBB1_1565483_at probeset were: 3.78 ± 0.42, 3.19 (3.01 - 5.42); 7.74 ± 0.13, 7.04 (5.1 - 11.7); and 8.27 ± 0.23, 8.37 (5.33 - 11.57), for control, standard risk and high-risk patients respectively. Expression values for the EGFR/ERBB1_1565484_x_at probeset were: 3.47 ± 0.38, 2.9 (2.81 - 4.95); 6.7 ± 0.1, 6.3 (4.47 - 10.15); and 7.05 ± 0.17, 7.07 (4.5 - 9.72), for control, standard-risk and high-risk patients respectively (Table S2).

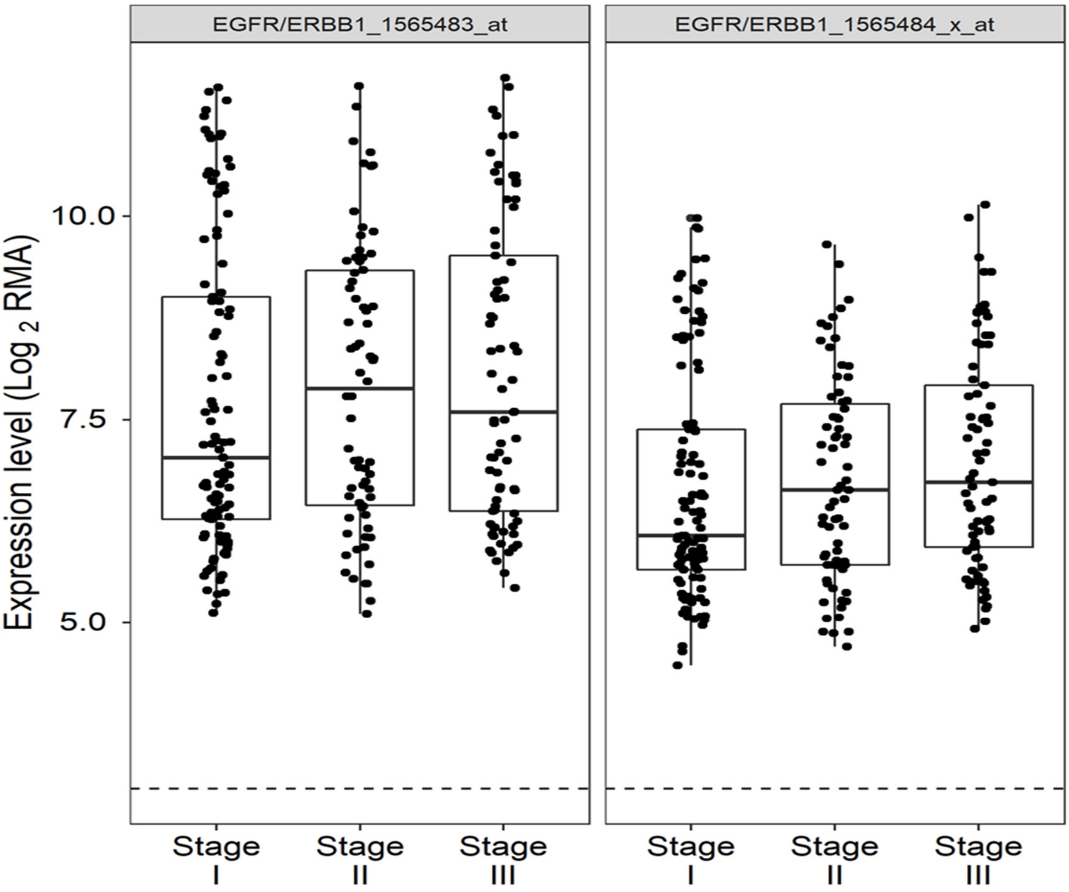

Figure S2. Absolute ERBB1 Gene Expression Levels in Malignant Plasma Cells from SKY-92 Risk-assessed MM Patients Categorized According to Prognostic ISS Staging Criteria. We examined the gene expression data in the archived MM dataset GSE19784 and the control plasma cell dataset from GSE171739. We examined normalized log_2 RMA expression values for the two ERBB1 probesets: EGFR/ERBB1_1565483_at and EGFR/ERBB1_1565484_x_at, interrogated from Stage I patients (N=109), Stage II MM patients (N=70) and Stage III MM patients (N=77). The gene expression levels for the 2 probesets (filled circles) are depicted in the box-plot that represents the median expression (horizontal line in the box), the 75th and 25th quantile box, and the whiskers for the 3rd quartile + 1.5*(interquartile range) and 1st quartile - 1.5*(interquartile range) of the expression values. We compared the expression values of the standard risk and high-risk MM samples with the upper 95% confidence interval for the lowest expressing 100 probesets obtained from 7 control samples (dashed line = 3.0; Mean = 2.89 ± 0.04). The EGFR/ERBB1 gene expression levels for both probesets were above the lowest expressing probesets in all MM samples. The lowest expression for the EGFR/ERBB1_1565484_x_at probeset obtained from Stage I patient was 4.47 (Table S3).

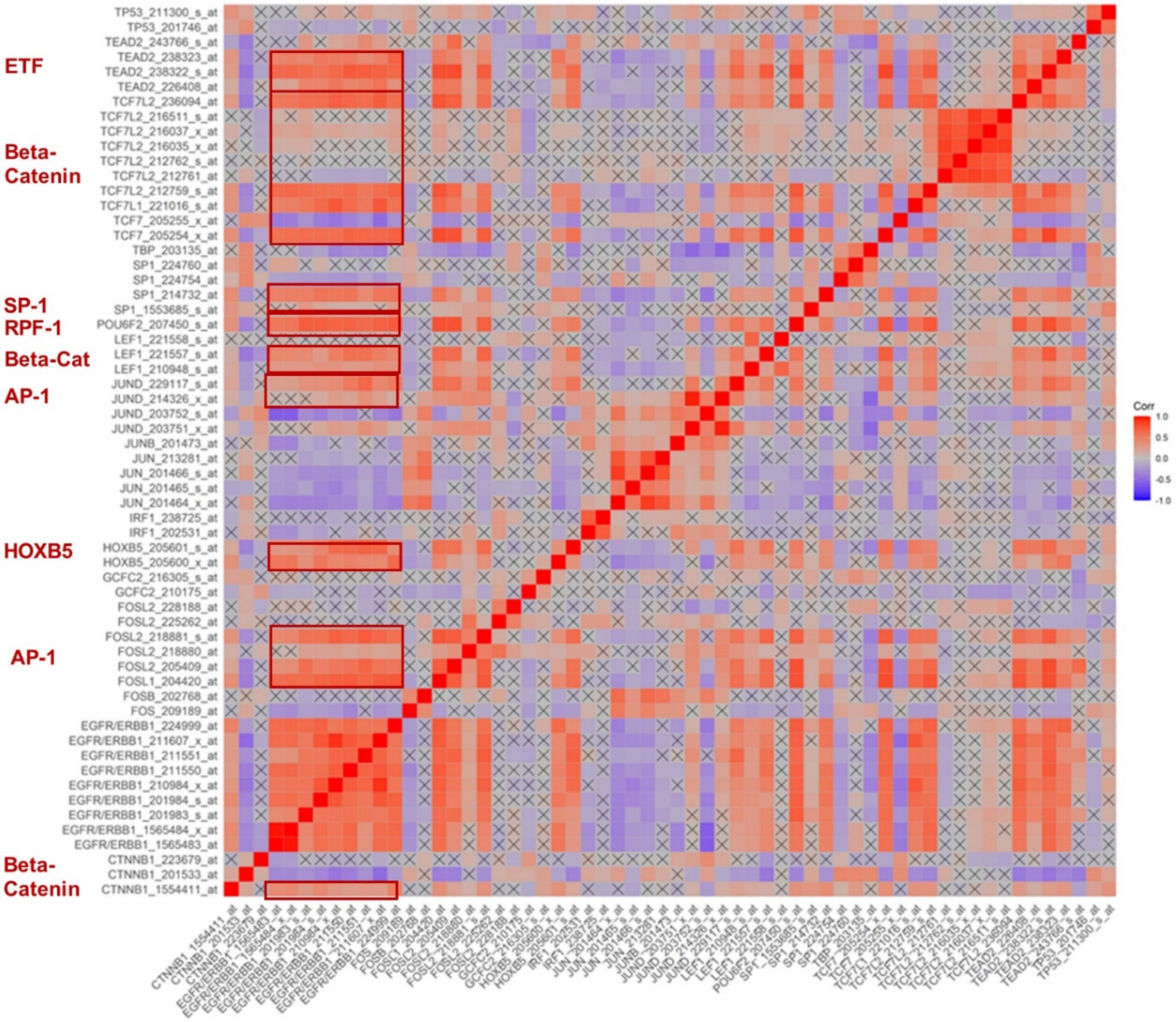

Figure S3. Correlation matrix of EGFR/ERBB1 probesets and transcription factors in MM patients. Pairwise Pearson correlations were performed between EGFR/ERBB1 and transcription factor probesets (60 probsets representing 21 genes) and the correlation coefficients calculated across 282 MM patients (GSE19784) (Corr) are depicted on the heatmap ranging from positive correlations (red) to negative correlations (blue) organized according to similarly expressed probesets. A total of 3600 pairwise correlations were formed of which 2302 were deemed to be significant (P<0.05 and FDR < 0.1; non-significant correlations are indicated with a black cross in the heat map). All 9 probesets for EGFR/ERRB1 formed a highly co-regulated cluster. The dark red boxes show co-regulated probesets for transcription factor binding mRNAs that bind to the promoter regions labelled with dark red text that were positively correlated with the EGFR/ERBB1 probesets. Three probesets represented mRNA coding for proteins (TEAD2) that bind to the ETF promoter; 6 probesets (2 for JUND, 3 for FOSL2, 1 for FOSL1) represented proteins that bind to the AP-1 promoter; 9 probesets represented proteins that bind to the Beta-Catenin TCF promoter (TCF7, TCF7L1, 4 probesets for TCF7L2 and CTNNB1, 2 probesets for LEF1); 2 probsets for the HOXB5 promoter; 2 probesets representing proteins that bind to the SP-1 promoter and POU6F2 representing protein that binds to the RPF-1 promoter were positively correlated with EGFR/ERRB1 expression.

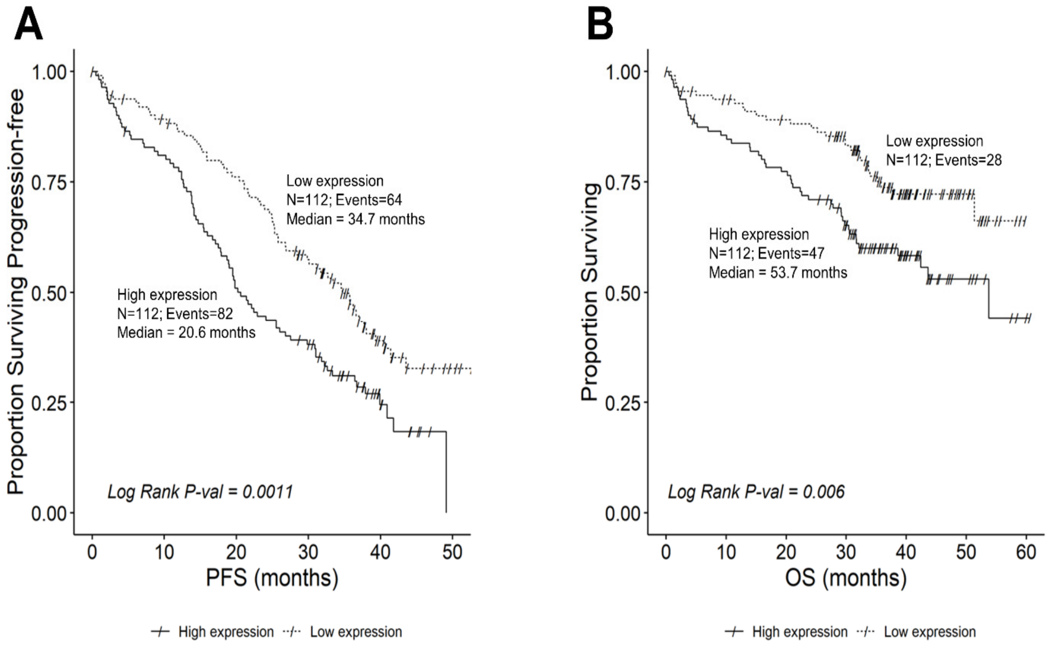

Figure S4. Higher ERBB1/EGFR Expression Level is Associated with Shorter PFS and OS in Newly Diagnosed MM patients. Archived PFS and OS data from newly diagnosed MM patients enrolled in the HOVON-65/GMMG-HD4 randomized clinical trial (ISRCTN64455289) was combined with mRNA expression of the ERBB1 probeset 1565484_x_at (GSE19784) to assess the PFS and OS times in these patients in relationship to EGFR/ERB1 expression. RMA normalized values from 282 newly diagnosed MM patients (pooled standard-risk and high-risk samples from GSE19784) were rank-ordered according to the transcript-level expression of EGFR/ERBB1 (Probeset: ERBB1_1565484_x_at). OS and PFS times were evaluable for 280 of these 282 MM patients. We compared the PFS (Panel A) and OS (Panel B) outcomes for MM patients with the highest expression level of ERBB1/EGFR (i.e., top 40% with the highest observed expression level; N=112) with the PFS and OS times for MM patients with the lowest expression level of ERBB1/EGFR (i.e., bottom 40% with the lowest observed expression level; N=112). [A] Patients with highest expression level of ERBB1/EGFR exhibited a significantly worse PFS outcome than patients with the lowest ERBB1/EGFR expression level (Log rank Chi-square value = 10.57, P-value = 0.0011). [B]. Patients with the highest expression level of ERBB1/EGFR exhibited a significantly worse OS outcome than patients with lowest ERBB1/EGFR expression level (Log rank Chi-square value = 7.68, P-value = 0.006).

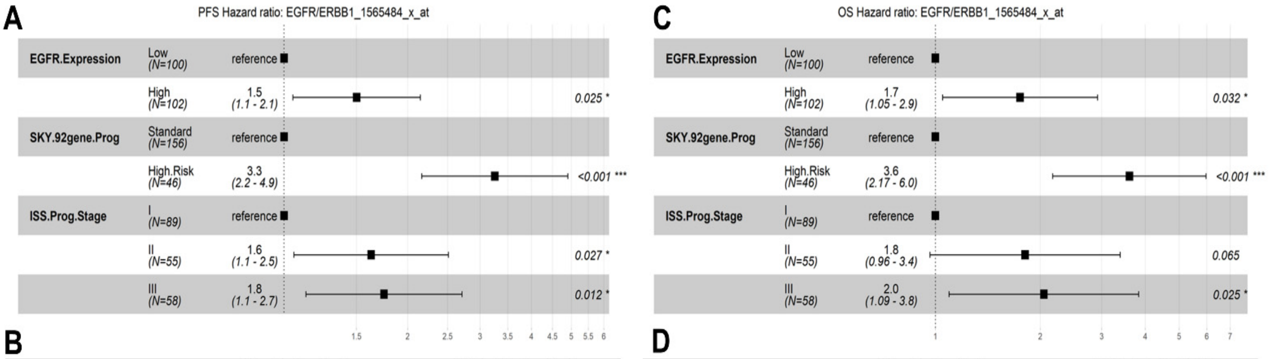

B

Factor	MM group	Univariate Cox Model HR (95% CI)	Univariate Cox Model P-value	Multivariate Cox Model HR (95% CI)	Multivariate Cox Model P-value
EGFR.Expression	High	1.85 (1.3 - 2.62)	0.00055	1.5 (1.05 - 2.15)	0.025
SKY.92gene.Prog	High Risk	4.12 (2.78 - 6.1)	< 0.00001	3.26 (2.16 - 4.9)	<0.001
ISS.Prog.Stage	II	1.96 (1.28 - 2.99)	0.00199	1.63 (1.06 - 2.51)	0.027
	III	2.43 (1.6 - 3.69)	0.00003	1.75 (1.13 - 2.71)	0.012

D

Factor	MM group	Univariate Cox Model HR (95% CI)	Univariate Cox Model P-value	Multivariate Cox Model HR (95% CI)	Multivariate Cox Model P-value
EGFR.Expression	High	2.28 (1.38 - 3.78)	0.00129	1.75 (1.05 - 2.92)	0.032
SKY.92gene.Prog	High Risk	4.82 (2.98 - 7.79)	< 0.00001	3.6 (2.17 - 5.97)	<0.001
ISS.Prog.Stage	II	2.41 (1.3 - 4.49)	0.00527	1.81 (0.96 - 3.39)	0.065
	III	3.23 (1.77 - 5.9)	0.00013	2.05 (1.09 - 3.83)	0.025

Figure S5. The unfavorable impact of higher EGFR/ERBB1 expression level on PFS and OS outcomes of MM patients in univariate and multivariate Cox proportional hazards models. Gene chip normalized expression values (Log_2 RMA) for the EGFR/ERBB1 probeset 1565484_x_at for MM samples (GSE19784) (high expression = top 40% with the highest observed expression level; N=112 of which 102 patients were evaluable; low expression = bottom 40% with the lowest observed expression level; N=112 of which 100 patients were evaluable), was correlated with PFS (Panels A&B) and OS outcomes (Panels C&D) in both univariate and multivariate Cox proportional hazards models that included the risk assignment based on the SKY-92 gene signature (High risk, N=46); Standard risk, N=156) and ISS stage (Stage I, N=89; Stage II, N=55; Stage III, N=58) as categorical covariates. Depicted are the Forest plots of HRs along with the corresponding tabulized values for the HRs and P-values for each covariate in univariate and multivariate Cox proportional hazards models.

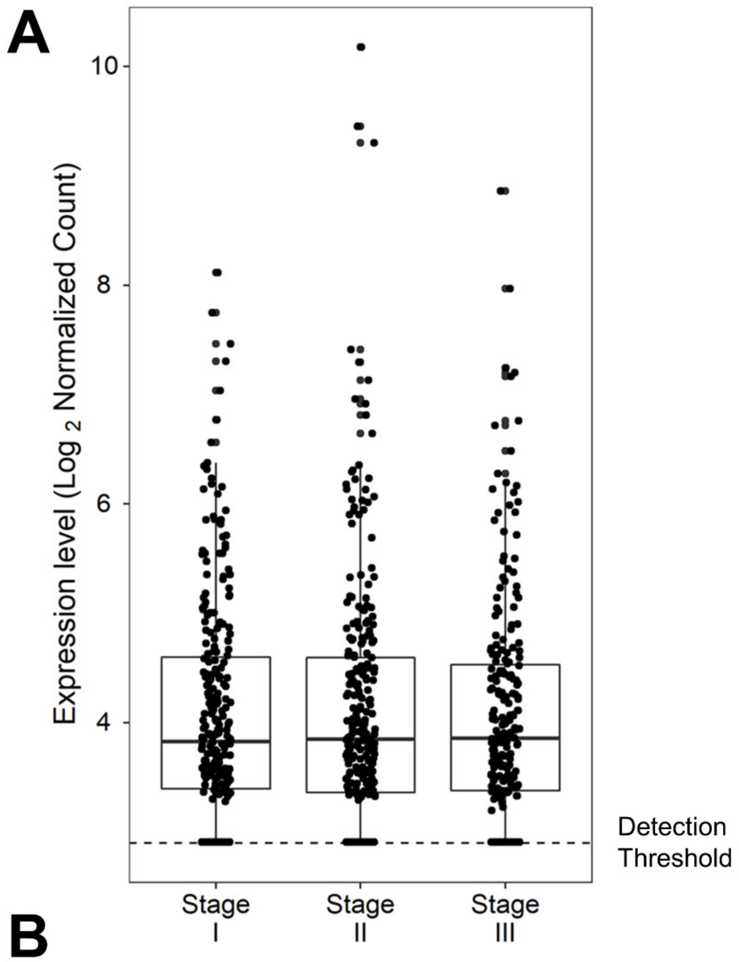

B

MM patient group ISS Stage	N	Mean ± SEM	Median (Range)	Comparison vs. Stage I Fold Change	Adj P-value
Stage I	267	4.07 ± 0.06	3.83 (2.91 - 8.12)		
Stage II	276	4.10 ± 0.07	3.85 (2.91 - 10.18)	1.29	0.89
Stage III	223	4.07 ± 0.07	3.86 (2.91 - 8.86)	0.98	0.80

Figure S6. Expression levels for EGFR/ERBB1 in malignant plasma cells from 766 newly diagnosed MM patients according to prognostic ISS stage. We examined the gene expression data in the archived MM dataset downloaded from the GDC portal: https://gdc.cancer.gov/about-gdc/contributed-genomic-data-cancer-research/foundation-medicine/multiple-myeloma-research-foundation-mmrf. We compared expression normalized $\log_2$ count values (variance stabilizing data transformation was performed to account for zero count data for undetected gene) for EGFR/ERBB1 comparing Stage I patients (N=267) to Stage II (N=276) and Stage III MM patients (N=223). [A] The expression of EGFR/ERBB1 (filled circles) is depicted in the box-plots representing the median expression (horizontal line in the box), the 75th and 25th quantile box, and the whiskers representing the 3rd quartile + 1.5*(interquartile range) and 1st quartile - 1.5*(interquartile range) of the expression values. We determined the lowest detection level for the normalized, variance stabilized $\log_2$ count value at which there were zero alignments to the gene (detection level = 2.91 represented by the dashed line). The number patients with zero counts (less than the 2.91 $\log_2$ transformed value) across all 3 stages for EGFR/ERBB1 expression was 157. [B] Pairwise comparisons for Stage II versus Stage I and Stage III versus Stage I (fold-change determined from $\log_2$ fold difference) was carried across groups using the normalization method in the DESeq2 algorithm (implemented in R) to calculate the Wald statistic and P-values correcting for multiple comparisons. No significant differences were observed for the pairwise comparisons.

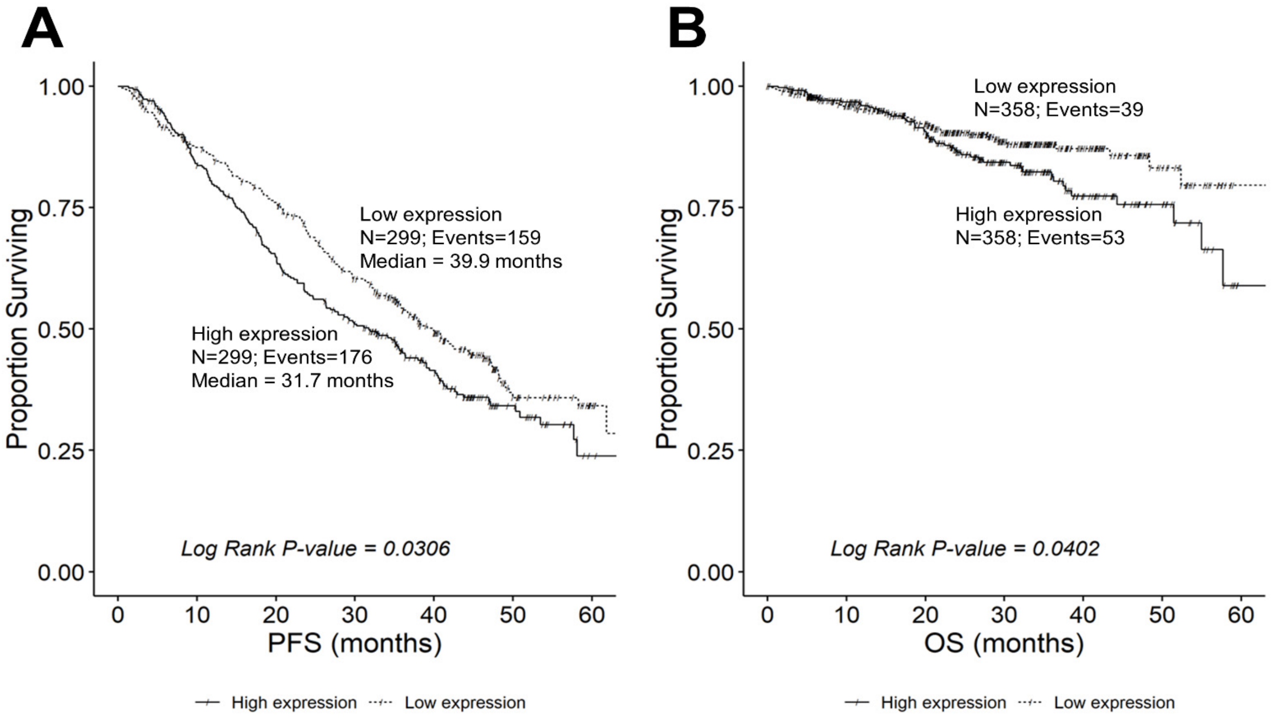

Figure S7. Higher ERBB1/EGFR Expression Level is Associated with Shorter PFS and OS in Newly Diagnosed MM patients from the MMRF-COMPASS study. Summarized RNA-seq datasets for 995 newly diagnosed Multiple Myeloma patients were down loaded from the GDC data portal: https://gdc.cancer.gov/about-gdc/contributed-genomic-data-cancer-research/foundation-medicine/multiple-myeloma-research-foundation-mmrf. The RNAseq data sets were combined with matched PFS (Panel A) and OS (Panel B) data. We compared high expression versus low expression values for normalized count data in "fragments per kilobase of transcript per million mapped reads upper quartile" (FPKM-UQ) aligned to the EGFR/ERBB1 sequence (GRCh38.p0 genome build). [**A**] The PFS curve depicts 598 evaluable cases for which disease progression was recorded. Patients with the highest expression level of ERBB1/EGFR (i.e., top 50% with the highest observed expression level; N = 299, Recorded events = 176; Median = 31.7 [95% CI: 26.25 - 38.59] years) showed a statistical difference compared to the PFS times for patients with the lowest expression level of ERBB1/EGFR (i.e., bottom 50% with the lowest observed expression level; N = 299, Recorded events = 159; 39.9 [95% CI: 35.43 - 47.24] years; Log Rank Chi Square = 4.67, P-value = 0.0306). [**B**] OS curves depict proportion of patients surviving cancer related deaths for 716 evaluable cases. Patients with the highest expression level of ERBB1/EGFR (i.e., top 50% with the highest observed expression level; N = 358, Recorded events = 53) showed a borderline statistical difference compared to the OS times for patients with the lowest expression level of ERBB1/EGFR (i.e., bottom 50% with the lowest observed expression level; N = 358, Recorded events = 39; Log Rank Chi Square = 4.21, P-value = 0.0402).
